# Diversity of Mycoviruses Present in Strains of Binucleate *Rhizoctonia* and Multinucleate *Rhizoctonia*, Causal Agents for Potato Stem Canker or Black Scurf

**DOI:** 10.3390/jof9020214

**Published:** 2023-02-06

**Authors:** Yuting Li, Naibo Yang, Tongyu Mu, Xuehong Wu, Can Zhao

**Affiliations:** 1 College of Plant Protection, China Agricultural University, Beijing 100193, China; 2 College of Plant Protection, Northeast Agricultural University, Harbin 150030, China; 3 College of Horticulture, China Agricultural University, Beijing 100193, China

**Keywords:** binucleate *Rhizoctonia*, multinucleate *Rhizoctonia*, metatranscriptome sequencing, mycoviral diversity, positive single-stranded RNA

## Abstract

In this study, the diversity of putative mycoviruses present in 66 strains of binucleate *Rhizoctonia* (BNR, including anastomosis group (AG)-A, AG-Fa, AG-K, and AG-W) and 192 strains of multinucleate *Rhizoctonia* (MNR, including AG-1-IA, AG-2-1, AG-3 PT, AG-4HGI, AG-4HGII, AG-4HGIII, and AG-5), which are the causal agents of potato stem canker or black scurf, was studied using metatranscriptome sequencing. The number of contigs related to mycoviruses identified from BNR and MNR was 173 and 485, respectively. On average, each strain of BNR accommodated 2.62 putative mycoviruses, while each strain of MNR accommodated 2.53 putative mycoviruses. Putative mycoviruses detected in both BNR and MNR contained positive single-stranded RNA (+ssRNA), double-stranded RNA (dsRNA), and negative single-stranded RNA (-ssRNA) genomes, with +ssRNA genome being the prevalent nucleic acid type (82.08% in BNR and 75.46% in MNR). Except for 3 unclassified, 170 putative mycoviruses found in BNR belonged to 13 families; excluding 33 unclassified, 452 putative mycoviruses found in MNR belonged to 19 families. Through genome organization, multiple alignments, and phylogenetic analyses, 4 new parititviruses, 39 novel mitoviruses, and 4 new hypoviruses with nearly whole genome were detected in the 258 strains of BNR and MNR.

## 1. Introduction

Mycovirus, first reported in 1960 [1], was proven widely distributing in various fungi and oomycetes [2,3,4]. Most mycoviruses infect latently, and a few of them could alter the phenotype of fungal hosts [5,6]. Mycoviruses associated with hypervirulence could increase the growth, sporulation capacity, and pathogenicity of their fungal hosts, such as Sclerotinia sclerotiorum hypovirus 2 (SsHV2) [7], Phytophthora infestans RNA virus 2 (PiRV-2) [8], and Colletotrichum higginsianum nonsegmented dsRNA virus 1 (ChNRV1) [9], which enhances the growth rate of *Monilinia fructicola* [7], sporulation capacity of *Phytophthora infestans* [8], and pathogenicity of *Colletotrichum higginsianum* [9], respectively. Conversely, mycoviruses associated with hypovirulence could attenuate growth, sporulation capacity, mycotoxin, and pathogenicity [10,11,12], which makes hypovirulent mycoviruses considered a good candidate of biological control agent. The best example for hypovirulence is the successful biological control of chestnut blight using the hypovirus Cryphonectria hypovirus 1 (CHV1), which has inspired scientists to explore hypoviruses in more plant fungal pathogens [13]. Moreover, it has been reported that spraying strain DT-8 containing Sclerotinia sclerotiorum hypovirulence-associated DNA virus 1 (SsHADV1) at the early flowering stage can decrease disease severity of rapeseed stem rot by 67.6% and increase yield by 14.9% [14].

It was documented that *Rhizoctonia* hosted more than 100 mycoviruses, and most of them were isolated from multinucleate *Rhizoctonia* (MNR, whose nuclei per cell are at least three) (including *R. solani* anastomosis group (AG)-1, AG-2, AG-3 PT, and AG-4) and belonged to 8 families, namely *Partitiviridae*, *Mitoviridae*, *Narnaviridae*, *Endornaviridae*, *Hypoviridae*, *Totiviridae*, *Bunyaviridae* and *Fusariviridae*, and a proposed family Bipartitiviridae [3,15,16,17,18,19,20]. Only twelve mycoviruses were found in binucleate *Rhizoctonia* (BNR, whose nuclei per cell are two) including AG-Ba (*R. fumigate*), AG-DΙ (*R. cerealis*), and AG-Bb (*R. oryzae-sativae*), and belonged to four families (*Partitiviridae*, *Megabirnaviridae*, *Endornaviridae,* and *Mitoviridae*) and a proposed family Bipartitiviridae [15,21,22,23,24,25,26].

However, the diversity of mycoviruses remains largely unknown and is greatly undervalued [27,28,29,30]. To date, only three studies were focused on the diversity of mycoviruses associated with *Rhizoctonia* [31,32,33]. The mycoviral diversity of 84 *R. solani* isolates (whose anastomosis groups or subgroups were unknown) collected from the United States of America (USA) was investigated. Twenty-seven mycoviruses with positive single-stranded RNA (+ssRNA), negative single-stranded RNA (-ssRNA), and double-stranded RNA (dsRNA) genomes belonging to nine families (*Ophioviridae*, *Bunyaviridae*, *Endornaviridae*, *Botourmiaviridae*, *Mitoviridae*, *Tymoviridae, Barnaviridae*, *Benyviridae,* and *Partitiviridae*) were found [31]. Forty-seven partial or complete viral unique RNA dependent-RNA polymerase (RdRp) sequences with a high prevalence of +ssRNA genome were obtained in eight strains of *R. solani* AG-2-2LP, which belonged to seven families (*Endornaviridae*, *Partitiviridae*, *Mitoviridae*, *Benyviridae*, *Deltaflexiviridae*, *Fusariviridae,* and *Hypoviridae*) and five proposed families (Basidionarnaviridae, Bipartitiviridae, Mycoalphaviridae, Mycophleboviridae, and Phlegiviridae) [22]. Ten mycovirus-related contigs composing five mycoviruses were identified in 43 *R. solani* AG-1-IA isolates causing rice sheath blight; among the five mycoviruses, two mycoviruses were unclassified and three mycoviruses with dsRNA and +ssRNA genomes belonged to the two families, *Partitiviridae* and *Hypoviridae* [33].

Potato stem canker or black scurf caused by *Rhizoctonia* is an economically important disease all over the world, leading to marketable yield losses of up to 30% [34]. It is widely known that *R. solani* AG-3 PT is the main and the most aggressive causal agent of stem canker or black scurf on potatoes [35,36,37,38,39]. In addition to AG-3 PT, AG-1, AG-2, AG-4, AG-5, AG-6, AG-7, AG-8, AG-9, AG-10, AG-11, AG-12, and AG-13 were also reported to cause Rhizoctonia disease on potato [35,36,39,40,41,42,43,44,45]. In addition, BNR, used to protect potatoes against MNR previously [46,47], was proven to cause slight symptoms of stem canker on potatoes, such as AG-A, AG-F, AG-G, AG-I, AG-K, AG-U, and AG-W [48,49,50,51,52].

Up to now, there are no reports focused on the diversity of mycoviruses associated with MNR causing potato Rhizoctonia diseases, to say nothing of the diversity of mycoviruses associated with BNR causing Rhizoctonia diseases on potatoes. In the present study, the diversity of mycoviruses present in 66 strains of BNR and 192 strains of MNR collected across China, which were the causal agents of potato stem canker or black scurf, were analyzed comprehensively and systematically by metatranscriptome sequencing. The results obtained in this study will provide a theoretical basis and data support for the genetic evolution of mycoviruses found in *Rhizoctonia* and the exploration of hypovirulent mycoviruses as biological control resources for controlling Rhizoctonia diseases on potato.

## 2. Materials and Methods

### 2.1. Fungal Strains

**Figure 1:**
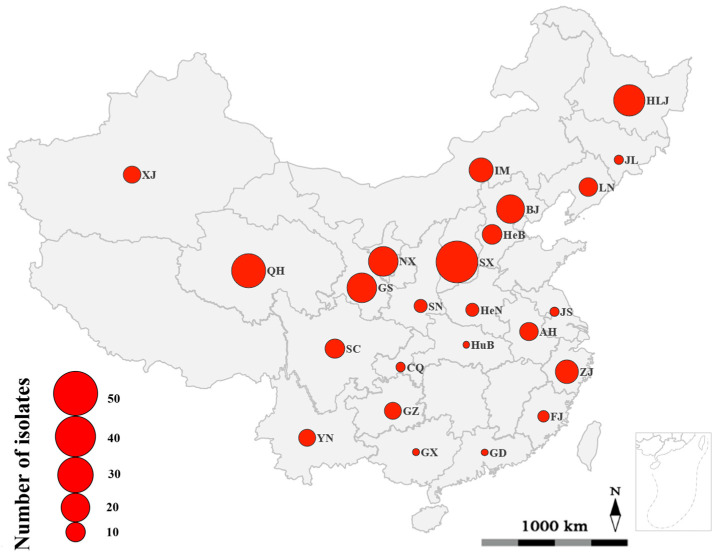
Geographic origins where *Rhizoctonia* isolates were collected. The size of the red spots represented the number of *Rhizoctonia* isolates collected from each province, municipality, or autonomous region. Abbreviation and full name of provinces, municipalities, and autonomous regions are as follows: AH, Anhui province; BJ, Beijing municipality; CQ, Chongqing municipality; FJ, Fujian province; GD, Guangdong province; GS, Gansu province; GX, Guangxi Zhuang autonomous region; GZ, Guizhou province; HeB, Hebei province; HeN, Henan province; HLJ, Heilongjiang province; HuB, Hubei province; IM, Inner Mongolia autonomous region; JL, Jilin province; JS, Jiangsu province; LN, Liaoning province; NX, Ningxia Hui autonomous region; QH, Qinghai province; SC, Sichuan province; SN, Shaanxi province; SX, Shanxi province; XJ, Xinjiang Uygur autonomous region; YN, Yunnan province; ZJ, Zhejiang province.

Sixty-six strains of BNR (identified as AG-A, AG-Fa, AG-K, and AG-W) and 192 strains of MNR (identified as AG-1-IB, AG-2-1, AG-3 PT, AG-4HGI, AG-4HGII, AG-4HGIII, and AG-5) were used in this study (Table S1), which were the causal agents of potato stem canker or black scurf. The 258 strains were collected from 18 provinces (Anhui, Fujian, Gansu, Guangdong, Guizhou, Hebei, Heilongjiang, Henan, Hubei, Jiangsu, Jilin, Liaoning, Qinghai, Shaanxi, Shanxi, Sichuan, Yunnan, and Zhejiang provinces), two municipalities (Beijing and Chongqing municipalities), and four autonomous regions (Guangxi Zhuang, Inner Mongolia, Ningxia Hui, and Xinjiang Uygur autonomous regions) across China (Figure 1). Among these 258 strains, 34 strains of BNR [48,53] and 95 strains of MNR [39] were reported in our previous studies [39,48,53], while the remaining 32 strains of BNR and the remaining 97 strains of MNR were identified in this study according to the methods described previously [39,48,53]. All 258 strains were cultured on potato dextrose agar (PDA) plates at 25 °C in the dark for five days prior to their use.

### 2.2. Extraction of Total RNA

For extracting total RNA, the 258 strains of BNR and MNR were cultured on PDA plates covered with cellophane film membranes (PDA-CF) at 25 °C in the dark for five days. Approximately 0.5 g of fresh mycelia were harvested from PDA-CF and ground to fine powder in liquid nitrogen, and then total RNA was extracted using TRIzol Reagent (Invitrogen, Carlsbad, CA, USA) according to the manufacturer’s protocol. The concentrations and quality of RNA samples were measured using an ultramicro spectrophotometer (Nanodrop 2000, Thermo Scientific, Waltham, MA, USA). To confirm the RNA integrity, 1.0% (*w*/*v*) gel electrophoresis agarose was used. Finally, RNA samples were pooled to have the same final concentration (~200 ng/µL), resulting in two pools which were from BNR and MNR, respectively.

### 2.3. Metatranscriptome Sequencing

Metatranscriptome sequencing of the 258 strains of BNR and MNR was conducted by Shanghai Biotechnology Corporation using an Illumina X-TEN instrument with paired-end program. TruSeq Stranded Total RNA LT Sample Prep Kit (Illumina, San Diego, CA, USA) was used to establish sequencing libraries of strains of BNR and MNR from rRNA-depleted total RNA. Library quality was checked using Qubit^®^ 2.0 Fluorometer (Invitrogen, Q32866) and Agilent Technologies 2100 Bioanalyzer (Agilent Technologies, Santa Clara, CA, USA). The low-quality reads were filtered out to obtain high-quality clean reads. De novo sequence assembly was constructed using CLC Genomics Workbench version 6.0.4 software. The resulting final sequences were subjected to National Center for the Biotechnology Information (NCBI) non-redundant (NR) database and aligned using BLASTx to confirm the mycovirus-like contigs and classification status, nucleic acid type, and the virus best matched of these contigs.

### 2.4. Genome Organization and Phylogenetic Analysis

NCBI ORF finder program (https://www.ncbi.nlm.nih.gov/orffinder/, accessed on 1 June 2022) was used to predict open reading frames (ORFs) of contigs related to mycoviruses obtained from metatranscriptome sequencing based on standard genetic code or fungal mitochondrial genetic code (whose number is 4). BLASTp and BLASTx were used to search for homologous mycoviruses against NCBI NR database. When the e-value is less than or equal to 1 × 10^−5^, the annotation result is considered reliable. Conserved Domain Database (CDD) (http://www.ncbi.nlm.nih.gov/Structure/cdd/wrpsb.cgi, accessed on 1 June 2022), Protein Family (Pfam) database (http://pfam.sanger.ac.uk/, accessed on 1 June 2022), and PROSITE database (http://www.expasy.ch/, accessed on 1 June 2022) were used to find conserved motifs of amino acid (aa) sequences of putative mycoviruses. The aa sequences alignments were conducted by CLUSTAL_X [54]. Phylogenetic trees based on aa sequences of RNA dependent RNA polymerase (RdRp), or polyprotein were conducted by the maximum likelihood (ML) method in Jones–Taylor–Thornton (JTT) model with 1000 bootstrap replicates using MEGA software version 6.0 [55].

### 2.5. Virus Names

The name of a novel putative mycovirus identified in this study for the first time is named according to a previous reference [56], which consists of three parts: (I) the first part of the name is the source of the virus; (II) the second part of the name shows the virus taxonomical group; and (III) the third part of the name is a progressive number [57]. For example, “Rhizoctonia solani [part I] partitivirus [part II] 12 [part III]” presents a new partitivirus and the twelfth partitivirus found in *R. solani*. A mycovirus previously reported, which was also identified in this study, was labeled with “BNR” or “MNR” to indicate its sources in this study. For example, “Rhizoctonia solani partitivirus 6-BNR” presents a strain of Rhizoctonia solani partitivirus 6 reported previously [58] and is identified from a strain of BNR in this study.

## 3. Results

### 3.1. Comparison of Mycoviral Diversity in BNR and MNR

Metatranscriptome sequencing was conducted based on the libraries of strains of BNR and MNR, and 11.34 GB raw data and 11.22 GB raw data were obtained from the libraries of strains of BNR and MNR, respectively. After filtering and de novo assembly, 99,951 contigs and 112,939 contigs were acquired from strains of BNR and MNR, respectively (Table 1). Homology of the contigs with more than 200 nt in length were conducted by screening against GenBank NR database, the most detailed protein database currently used for protein function and structure annotation. The best matches of these contigs were determined based on the top hit (the highest identity and query cover to the available viral genomes in the NR database) from BLASTx and e-values (≤1 × 10^−5^). As a result, a total of 658 contigs were best matched with viral genomes, with 173 contigs and 485 contigs being found in BNR and MNR, respectively. Annotation results showed that 472 (71.7%) out of these 658 contigs had low identities (≤70%) of aa sequences with mycoviruses reported previously. Contig ids, length, best match (most closely related viruses), query cover, amino acid identity, and e-value were listed in Table S2.

The results of BLASTx analysis showed that three and 33 contigs related to viruses obtained from BNR and MNR, respectively, were best matched with unclassified viruses reported previously and considered as putative unclassified mycoviruses. For example, First_Contig8786 with 1736 nt in length was most closely related to an unclassified virus, soybean leaf-associated negative-stranded RNA virus 4. The remaining 170 putative mycoviruses found in BNR and the remaining 452 putative mycoviruses found in MNR belonged to 13 and 19 virus families, respectively. The 11 virus families found in both BNR and MNR were *Benyviridae*, *Botourmiaviridae*, *Endornaviridae*, *Fusariviridae*, *Hypoviridae*, *Mitoviridae*, *Narnaviridae*, *Partitiviridae*, *Totiviridae*, *Mymonaviridae,* and a proposed family Bipartitiviridae. Moreover, the two families (*Virgaviridae* and *Rhabdoviridae*) were only detected in BNR, while the eight families (*Betaflexiviridae*, *Bunyaviridae*, *Deltaflexiviridae*, *Gammaflexiviridae*, *Megabirnaviridae*, *Togaviridae*, *Tombusviridae,* and *Tymoviridae*) were only discovered in MNR (Table 2).

Double-stranded RNA, +ssRNA, and -ssRNA is the main nucleic acid types assembling the mycovirome of BNR and MNR. The proportion of these three nucleic acid types in BNR and MNR was similar. The proportion of putative mycoviruses with +ssRNA genome found in both BNR (142 putative mycoviruses, 82.08%) and MNR (366 putative mycoviruses, 75.46%) was the highest, followed by dsRNA putative mycoviruses (13.87% in BNR; 17.73% in MNR), and -ssRNA putative mycoviruses (4.04% in BNR; 6.81% in MNR).

### 3.2. Genome Organization and Phylogenetic Analysis of Putative Members of the Family Partitiviridae

**Figure 2:**
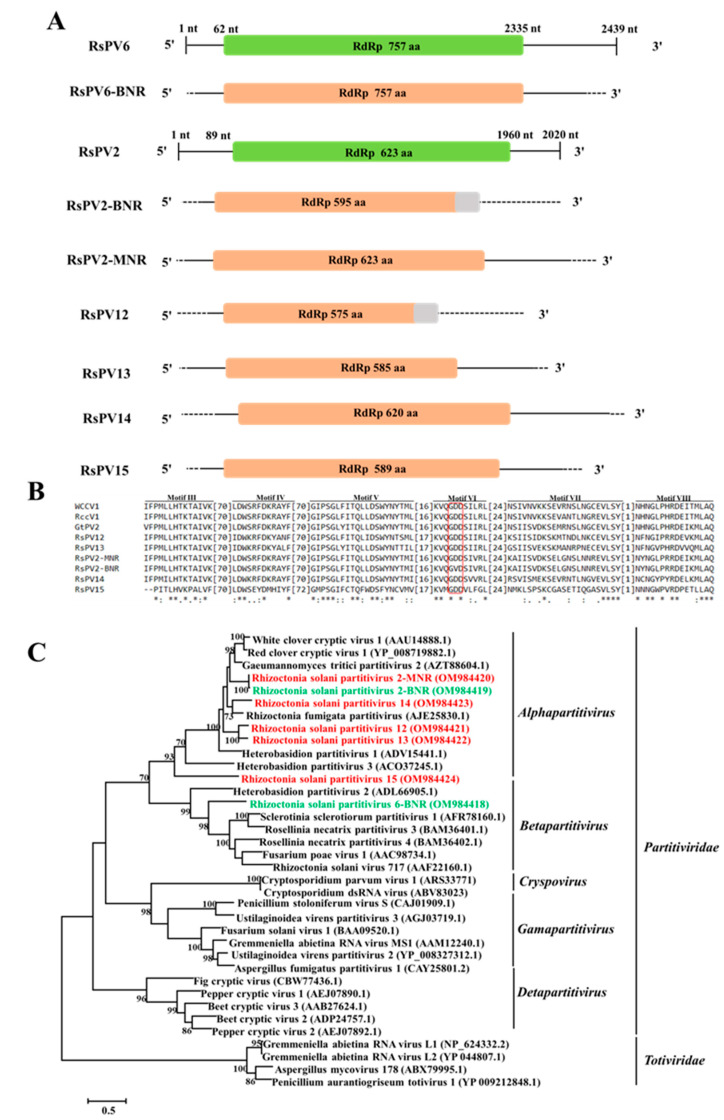
The genome organization, multiple alignments of amino acid (aa) sequences, and phylogenetic analysis of partitiviruses found in binucleate *Rhizcotonia* (BNR) and multinucleate *Rhizcotonia* (MNR). (**A**) Schematic diagrams of genome organization of the seven partitiviruses found in this study, namely Rhizoctonia solani partitivirus 6-BNR (RsPV6-BNR), Rhizoctonia solani parititvirus 2-BNR (RsPV2-BNR), RsPV2-MNR, Rhizoctonia solani partitivirus 12–15 (RsPV12–15), and the two reference partitiviruses reported previously, namely, Rhizoctonia solani partitivirus 6 (RsPV6) and Rhizoctonia solani parititvirus 2 (RsPV2). Dotted lines represent the undetermined untranslated regions (UTRs). The orange rectangles and the grey rectangles were used to represent the obtained ORFs and undetermined 3’-ORFs in the schematic diagrams, respectively. Full name, abbreviation, and GenBank accession number of the two reference viruses are as follows: Rhizoctonia solani partitivirus 2 (RsPV2, NC_023684.1), Rhizoctonia solani partitivirus 6 (RsPV6, MK809397.1). (**B**) Multiple alignments of aa sequences of RNA dependent RNA polymerase (RdRp) of partitiviruses found in BNR, MNR, and three reference viruses. Asterisks, colons, and dots represent identical, conserved, and semi-conserved aa residues, respectively. Red box indicates the highly conserved GDD tripeptide. Full name, abbreviation, and GenBank accession number of the three reference viruses are as follows: White clover cryptic virus 1 (WCCV1, AAU14888.1), Red clover cryptic virus 1 (RccV1, YP_008719882.1), Gaeumannomyces tritici partitivirus 2 (GtPV2, AZT88604.1). (**C**) Phylogenetic tree based on aa sequences of RdRp of partitiviruses found in BNR, MNR, and reference viruses. Green font indicates partitiviruses found in BNR and red font indicates partitiviruses found in MNR. Full name and GenBank accession number of reference viruses are shown.

Ten and sixty-four contigs related to mycoviruses belonging to the family *Partitiviridae* were obtained in BNR and MNR, respectively. Among the seventy-four contigs, seven contigs (two from BNR and five from MNR) whose lengths are longer than 1.8 kbp (Figure 2A) were selected (Table 3) to perform genome organization and phylogenetic analysis. The contig ids, best matches, names, and GenBank accession numbers of the seven partitiviruses were listed in Table 3. Contig5377 and Contig47566, found in BNR, were most closely related to the aa sequence of RdRp of Rhizoctonia solani partitivirus 6 (RsPV6, 99.08%) [58] and Rhizoctonia solani partitivirus 2 (RsPV2, 99.66%) [56], respectively, which were named RsPV6-BNR and RsPV2-BNR, respectively. Contig7211, found in MNR, were most closely related to aa sequence of RdRp of RsPV2 (99.84%) [45] and named RsPV2-MNR. Contig6469, Contig8296, Contig25890, and Contig59663, found in MNR, were most closely related to the aa sequence of RdRp of Ceratobasidium partitivirus (56.03%) [59], Rhizoctonia solani partitivirus 3 (53.51%) [60], Rhizoctonia fumigata partitivirus (62.68%) [24], and Raphanus sativus cryptic virus 1 (56.57%) [61], respectively, which were designated as Rhizoctonia solani partitivirus 12, 13, 14, and 15 (RsPV12–15), respectively. RsPV12–15 are new species of the family *Partitiviridae* according to the classification criteria of the family *Partitiviridae* provided by International Committee on Taxonomy of Viruses (ICTV, https://ictv.global/report/chapter/partitiviridae/partitiviridae, accessed on 1 June 2022).

Multiple alignments of aa sequences of RdRp of these seven partitiviruses found in BNR and MNR, and their homologous viruses were performed, and six conserved motifs (motif III–VIII) were revealed in the viral RdRp domain. Furthermore, GDD tripeptide (the hallmark of most viral RdRps) was found in motif VI (Figure 2B). Phylogenetic analysis showed that RsPV6-BNR clustered together with members of the genus *Betapartitivirus*, while RsPV2-BNR, RsPV2-MNR, and RsPV12–15 were most closely related to members of the genus *Alphapartitivirus* (Figure 2C).

### 3.3. Genome Organization and Phylogenetic Analysis of Putative Members of the Family Mitoviridae

The 78 and 149 contigs related to mycoviruses associated with the family *Mitoviridae* were found in BNR and MNR, respectively. Among the 227 contigs, 54 contigs (26 contigs found in BNR and 28 contigs found in MNR) whose lengths are longer than 3000 nt or which can encode complete RdRp was chosen to predict open reading frame (ORF) and perform multiple alignments. The contig ids, best matches, names, and GenBank accession numbers of the 54 mitoviruses were listed in Table 4. The results showed that among the 54 contigs, the lengths of aa sequences of 50 contigs (92.59%) were over 700 aa, and the lengths of aa sequences of 21 contigs (38.89%) were over 900 aa. Since the lengths of complete aa sequence of RdRp of most mitoviruses submitted to the NCBI NR database (https://www.ncbi.nlm.nih.gov/protein, accessed on 1 June 2022) range from 500 aa to 900 aa, the lengths of aa sequences of RdRp of these 54 mitoviruses in this study are relatively longer than that of most mitoviruses reported previously.

**Figure 3:**
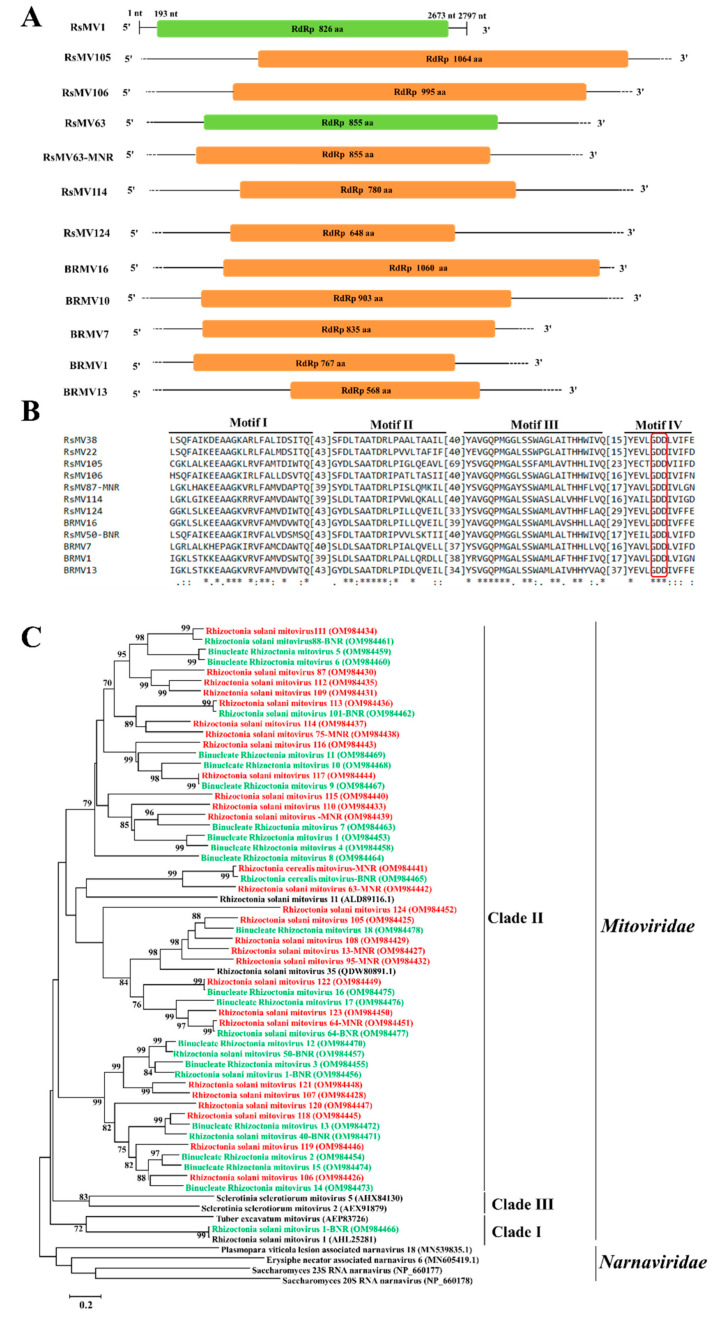
Genome organization, multiple alignments, and phylogenetic analysis of mitoviruses found in binucleate *Rhizoctonia* (BNR) and multinucleate *Rhizoctonia* (MNR). (**A**) Schematic diagrams of genome organization of the ten mitoviruses found in this study, namely Rhizoctonia solani mitovirus 105, 106, 114, 124 (RsMV105, 106, 114, 124), Rhizoctonia solani mitovirus 63-MNR, Binucleate Rhizoctonia mitovirus 1, 7, 10, 13, 16 (BRMV1,7,10, 13,16), and the two reference mitoviruses reported previously, namely, Rhizoctonia solani mitovirus 1 (RsMV1) and Rhizoctonia solani mitovirus 63 (RsMV63). Dotted lines represent the undetermined untranslated regions (UTRs). Full name, abbreviation, and GenBank accession number of the two reference viruses are as follows: Rhizoctonia solani mitovirus 1 (RsMV1, KC792591), Rhizoctonia solani mitovirus 63 (RsMV63, MZ043948.1). (**B**) Multiple alignments of amino acid (aa) sequence of RNA-dependent RNA polymerase (RdRp) of mitoviruses found in BNR, MNR, and two reference viruses. Asterisks, colons, and dots represent identical, conserved, and semi-conserved aa residues, respectively. Red box indicates the highly conserved GDD tripeptide. Full name, abbreviation, and GenBank accession number of reference the two viruses are as follows: Rhizoctonia solani mitoviruses 38 (RsMV38, QDW65426), Rhizoctonia solani mitoviruses 22 (RsMV22, QDW80890). (**C**) Phylogenetic analysis based on aa sequences of RdRp of mitoviruses found in BNR and MNR. Green font indicates mitoviruses found in BNR and red font indicates mitoviruses found in MNR. Full name and GenBank accession number of reference viruses are shown.

Among these fifty-four contigs, the length of aa sequence of four contigs (one from BNR and three from MNR) was shorter than 700 aa, the length of aa sequence of nine contigs (seven from BNR and two from MNR) ranged from 700 aa to 800 aa, the length of aa sequence of twenty contigs (eight from BNR and 12 from MNR) ranged from 800 aa to 900 aa, the length of aa sequence of twelve contigs (six from BNR and six from MNR) ranged from 900 aa to 1000 aa, and the length of aa sequence of nine contigs (four from BNR and five from MNR) were longer than 1000 aa. Ten contigs with these five length ranges (five contigs from BNR and five contigs from MNR) were chosen (Table 4) and used for genome organization and multiple alignments (Figure 3). All the ten mitoviruses contained an ORF encoding RdRp (Figure 3A) based on mitochondrial codon usage. Through multiple alignments of aa sequences of RdRp of these ten mitoviruses and their homologous viruses, four conserved motifs (motif III–VIII) were found (Figure 3B) in the viral RdRp domain; moreover, GDD tripeptide was found in motif IV (Figure 3B).

Phylogenetic analysis based on the aa sequences of RdRp showed that these 54 putative mycoviruses with nearly the whole genome found in BNR and MNR clustered into the family *Mitoviridae*. Except for contig2511 (Rhizoctonia solani mitovirus 1-BNR) which was clustered in clade I (Figure 3C), the remaining 53 contigs clustered together in clade II. Red fonts and green fonts indicate the mitoviruses found in BNR and MNR, respectively. A total of 38 (18 found in BNR and 20 found in MNR) out of the 54 mitoviruses discovered in this study are new species of the family *Mitoviridae* (Table 4).

### 3.4. Genome Organization and Phylogenetic Analysis of Putative Members of the Family Hypoviridae

One contig found in BNR and three contigs found in MNR with >8000 nt in length or encoding complete ORF were most closely related to hypoviruses, which were named Binucleate Rhizoctonia hypovirus 1 (BRHV1), Rhizoctonia solani hypovirus 9 (RsHV9), Rhizoctonia solani hypovirus 10 (RsHV10), and Rhizoctonia solani hypovirus 11 (RsHV11), respectively. The contig ids, best matches, names, and GenBank accession numbers of the four hypoviruses were listed in Table 5.

**Figure 4:**
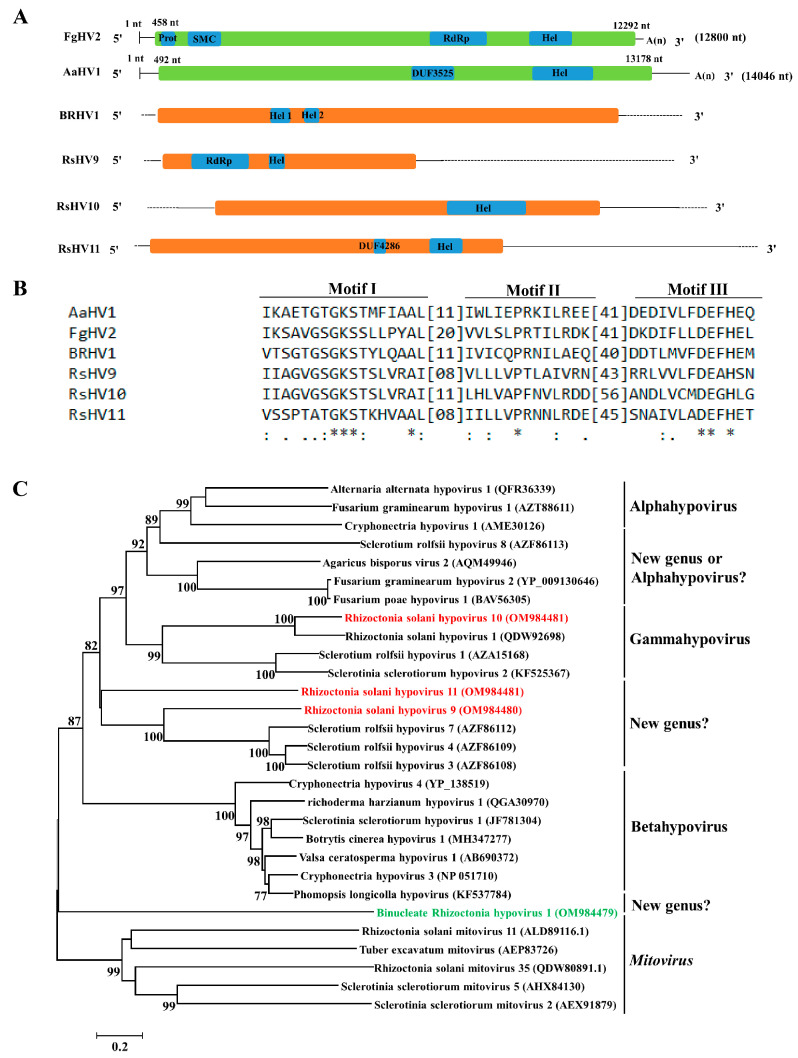
Genome organization, multiple alignments, and phylogenetic analysis of hypoviruses found in binucleate *Rhizoctonia* (BNR) and multinucleate *Rhizoctonia* (MNR). (**A**) Schematic diagrams of genome organization of the four hypoviruses found in this study, namely Binucleate Rhizoctonia hypovirus 1 (BRHV1), Rhizoctonia solani hypovirus 9–11 (RsHV9–11), and the two reference viruses, namely Fusarium graminearum Hypovirus 2 (FgHV2) and Alternaria alternata hypovirus 1 (AaHV1). Dotted lines represent the undetermined untranslated regions (UTRs). Full name, abbreviation, and GenBank accession number of the two reference viruses are as follows: Fusarium graminearum Hypovirus 2 (FgHV2, YP_009130646), Alternaria alternata hypovirus 1 (AaHV1, QFR36339). (**B**) Multiple alignments of amino acid (aa) of sequence of helicase (Hel) of hypoviruses found in BNR, MNR, and two reference viruses. Asterisks, colons, and dots represent identical, conserved, and semi-conserved aa residues, respectively. Full name, abbreviation, and GenBank accession number of the two reference viruses are as follows: Alternaria alternata hypovirus 1 (AaHV1, QFR36339), Fusarium graminearum hypovirus 2 (FgHV2, YP_009130646). (**C**) Phylogenetic analysis based on the aa sequences of polyprotein of hypoviruses found in BNR, MNR, and reference viruses. Green font indicates hypoviruses found in BNR and red font indicates hypoviruses found in MNR. Full name and GenBank accession number of reference viruses are shown.

Analysis of genome organization showed that these four hypoviruses all contained one +ssRNA encoding polyprotein (Figure 4A). The polyprotein of RsHV9 contained RdRp and RNA helicase (Hel) domains. The polyproteins of RsHV10 and BRHV1 contained one and two Hel domains, respectively. The polyprotein of RsHV11 contained DUF4286 and Hel domains. The Hel domain was found in the polyprotein of all the four hypoviruses, while the RdRp, necessary for mycovirus replication, was only contained in polyprotein of RsHV9. The similar characteristic had been documented in other members of the family *Hypoviridae*; for example, the RdRp domain was contained in polyprotein of Fusarium graminearum hypovirus 2 (FgHV2) [21] but was not contained in polyprotein of Alternaria alternata hypovirus 1 (AaHV1) [62]. Multiple alignment of aa sequences of Hel of the four hypoviruses and their homologous viruses revealed that three conserved motifs (motif I–III) (Figure 4B) were found in the Hel domain.

Phylogenetic analysis based on the aa sequences of polyprotein of the four hypoviruses and their homologous viruses showed that RsHV10 clustered into the genus Gammahypovirus, and RsHV9 and RsHV11 clustered together with unclassified hypoviruses, such as Sclerotium rolfsii hypovirus 3, 4, and 7 [63]. BRHV1 clustered into the family *Hypoviridae*, but BRHV1 was relatively distinct from other hypoviruses within the family *Hypoviridae* (Figure 4C).

## 4. Discussion

Our study represents in detail the first record of the putative mycoviruses associated with strains of BNR and MNR causing potato stem canker or black scurf in China and perhaps worldwide using metatranscriptome sequencing. In this study, putative mycoviruses belonging to eight families (*Benyviridae*, *Botourmiaviridae*, *Fusariviridae*, *Hypoviridae*, *Mymonaviridae*, *Rhabdoviridae*, *Totiviridae*, and *Virgaviridae*) and seven families (*Betaflexiviridae*, *Botourmiaviridae*, *Deltaflexiviridae*, *Gammaflexiviridae*, *Mymonaviridae*, *Togaviridae*, and *Tombusviridae*) were firstly found in BNR and MNR, respectively.

Metatranscriptome sequencing is widely used to discover mycoviruses in different host species and has supported the progress of research in virus pathogenesis and controlling of related diseases [33]; however, the lengths of some contigs related to mycoviruses obtained from metatranscriptome sequencing were less than 1000 nt, some of which might only cover 1% of the whole genome of the corresponding mycoviruses. In this study, there were 60 endornaviruses (84.51%) with less than 1000 nt in length found in MNR, and the shortest length of one endornavirus found in MNR is 209 nt, which indicated that most contigs related to endornaviruses found in MNR could not be used to predict a complete ORF. This might confuse our understanding of the diversity of mycoviruses associated with MNR. To weaken this confusion, it is necessary to assess the optimal number of strains used to establish sequencing libraries and thus ensure the integrity of sequencing results.

The family *Partitiviridae* contains five genera, namely *Alphapartitivirus*, *Betapartitivirus*, *Gammapartitivirus*, *Deltapartitivirus*, and *Cryspovirus* [19,64]. Recently, more and more unclassified partitiviruses previously were identified and proposed to be clustered into two new genera, Epsilonpartitivirus and Zetapartitivirus. For instance, Hubei partiti-like virus 11 [61], Hubei partiti-like virus 5 [61], and Hubei partiti-like virus 10 [61] belonged to the proposed genus Epsilonpartitivirus; Colletotrichum acutatum RNA virus 1 [65] and Aspergillus flavus partitivirus 1 [66] belonged to the proposed genus Zetapartitivirus. In addition, three partitiviruses, Aspergillus fumigatus partitivirus 2 [67], Alternaria alternata partitivirus 1 [68], and Delitschia confertaspora partitivirus 1 [69], were still unclassified. All the studies mentioned above indicated the diversity of mycoviruses within the family *Partitiviridae* was rich and evolutionary relationship between members of the family *Partitiviridae* was complex. In this study, 10 and 64 putative partitiviruses were found in BNR and MNR, respectively, accounting for 41.67% and 75.29% of putative dsRNA mycoviruses found in BNR and MNR. Among them, two partitiviruses found in BNR and five partitiviruses found in MNR had whole ORF and belonged to the two genera, *Alphapartitivirus* (RsPV2-BNR, RsPV2-MNR, and RsPV12–15) and *Betapartitivirus* (RsPV6-BNR). Especially, RsPV12–15 were new species of the genus *Alphapartitivirus*.

The family *Mitoviridae* was newly established according to the ICTV report 2019, which contained a single +ssRNA genome with approximately 3000 nt in length, and could not assemble virus particles [70,71]. Phylogenetic analysis showed that mitoviruses clustered into three clades (clades I, II, and III), and most mitoviruses discovered in *Rhizoctonia* clustered into clade II, such as Rhizoctonia solani mitovirus 2, Rhizoctonia solani mitovirus 11, Rhizoctonia solani mitovirus 22, Rhizoctonia solani mitovirus 34, and Rhizoctonia solani mitovirus 35. In this study, 26 and 28 mitoviruses whose lengths are longer than 3000 nt or which can encode complete RdRp were found in BNR and MNR, respectively. Except for Rhizoctonia solani mitovirus 1-BNR (Contig2511) which was clustered into clade I, the remaining 53 putative mitoviruses were clustered into clade II. Collectively, mitoviruses found in *Rhizoctonia* might evolve together with their host fungi, and thus major sequence divergence in mitoviral genomes might not be induced [20,72].

The family *Hypoviridae* was proposed to contain three genera, namely Alphahypovirus, Betahypovirus, and Gamahypovirus [73,74], and most hypoviruses could be classified into these three proposed genera. However, some mycoviruses, such as Sclerotium rolfsii hypovirus 3, Sclerotium rolfsii hypovirus 4, Sclerotium rolfsii hypovirus 7 [63], Fusarium graminearum hypovirus 2 [22], and Fusarium poae hypovirus 1 [75], could not be classified into any of these three proposed genera, which were clustered into three other clades (Figure 4C) [62]. In the present study, four novel hypoviruses (BRHV1, RsHV9, RsHV10, and RsHV11) were identified; among them, RsHV10 belongs to the proposed genus Gammahypovirus, but the remaining three hypoviruses (BRHV1, RsHV9, and RsHV11) cannot be clustered into any of the three proposed genera mentioned above. RsHV9 and RsHV11 clustered into the same clade, and BRHV1 clustered into another clade (Figure 4C). Therefore, three other new genera, such as “Deltahypovirus”, “Epsilonhypoviurs”, and “Zetahypovirus”, might be proposed to be established to accommodate these newly discovered hypoviruses.

Many members of the family *Hypoviridae* could affect the phenotypes of hosts, especially for decreasing the virulence, which made hypoviruses be considered potential biocontrol agents [76,77,78,79]. Cryphonectria hypovirus 1 (CHV1) was the most successfully applied hypovirus to control chestnut blight in Europe and USA [77]. Additionally, Cryphonectria hypovirus 2 (CHV2), Cryphonectria hypovirus 3 (CHV3), Botrytis cinerea hypovirus 1 (BcHV1), Fusarium graminearum hypovirus 2 (FgHV2), and Sclerotinia sclerotiorum hypovirus 2 (SsHV2) were proven to confer hypovirulence [21,73,74,77,78,79]. However, Cryphonectria hypovirus 4 (CHV4), Fusarium graminearum hypovirus 1 (FgHV1), Sclerotinia sclerotiorum hypovirus 1/SZ150 (SsHV1/SZ150), and Valsa ceratosperma hypovirus 1 (VcHV1) were recorded to be latent infection [80,81,82]. Whether the four novel hypoviruses (BRHV1, RsHV9, RsHV10, and RsHV11) found in this study can confer hypovirulence on their host fungi or not needs to be further studied and analyzed.

Our study expands the acknowledgment of the diversity of mycoviruses present in BNR and MNR and provides the resources for investigating the evolutionary relationship of mycoviruses detected in BNR and MNR. The influence of mycoviruses found in this study on their hosts, and the interactions of mycoviruses and their host fungi need to be studied further.

## Figures and Tables

**Table 1:** Comparison of metatranscriptome sequencing data and their annotation results of binucleate *Rhizoctonia* (BNR) and multinucleate *Rhizoctonia* (MNR).

Library Sample	Number of Contigs	Average Length (Base Pair)	Annotation		
			Host Fungi	Virus	Not Found
BNR	99,951	473.87	82,400 (82.44%)	225 (0.23%)	17,326 (17.33%)
MNR	112,939	477.17	94,251 (83.45%)	571 (0.51%)	18,117 (16.04%)

**Table 2:** Comparison of taxonomic status of putative mycoviruses found in binucleate *Rhizoctonia* (BNR) and multinucleate *Rhizoctonia* (MNR).

Type of Nucleic Acid	Virus Family	Number of Mycovirus Contigs	
		BNR	MNR
Positive single-stranded RNA (+ssRNA)	*Benyviridae*	14	15
	*Botourmiaviridae*	8	11
	*Endornaviridae*	21	71
	*Fusariviridae*	13	67
	*Hypoviridae*	5	20
	*Mitoviridae*	75	149
	*Narnaviridae*	4	10
	*Betaflexiviridae*	- ^a^	1
	*Deltaflexiviridae*	- ^a^	1
	*Gammaflexiviridae*	- ^a^	1
	*Tymoviridae*	- ^a^	13
	*Togaviridae*	- ^a^	3
	*Tombusviridae*	- ^a^	1
	*Virgaviridae*	1	- ^b^
	unclassified	1	5
Double-stranded RNA (dsRNA)	Bipartitiviridae	11	8
	*Partitiviridae*	10	64
	*Totiviridae*	3	11
	*Megabirnaviridae*	- ^a^	2
Negative single-stranded RNA (-ssRNA)	*Mymonaviridae*	4	1
	*Bunyaviridae*	- ^a^	3
	*Rhabdoviridae*	1	- ^b^
	unclassified	2	28
Total		173	485

Note: “^a^” indicated that no virus belonging to the eight families *Betaflexiviridae*, *Deltaflexiviridae*, *Gammaflexiviridae*, *Tymoviridae*, *Togaviridae*, *Tombusviridae*, *Megabirnaviridae,* and *Bunyaviridae* was detected in BNR; “^b^” indicated that no virus belonging to the two families *Virgaviridae* and *Rhabdoviridae* was detected in MNR.

**Table 3:** The information of two contigs found in binucleate *Rhizoctonia* (BNR) and five contigs found in multinucleate *Rhizoctonia* (MNR) related to RNA dependent RNA polymerase of mycoviruses associated with the family *Partitiviridae*.

Contig	Size (Amino Acid)	Name	Origin	Best Match	Identity	Query Cover	E-Value	Accession Number
Contig5377	757	Rhizoctonia solani partitivirus 6-BNR	BNR	Rhizoctonia solani partitivirus 6	99%	93%	0	OM984418
Contig47566	595	Rhizoctonia solani Partitivirus 2-BNR	BNR	Rhizoctonia solani partitivirus 2	99%	96%	0	OM984419
Contig7211	623	Rhizoctonia solani Partitivirus 2-MNR	MNR	Rhizoctonia solani partitivirus 2	99%	95%	0	OM984420
Contig6469 ^a^	575	Rhizoctonia solani Partitivirus 12	MNR	Ceratobasidium partitivirus	56%	95%	0	OM984421
Contig8296 ^a^	585	Rhizoctonia solani Partitivirus 13	MNR	Rhizoctonia solani partitivirus 3	53%	92%	0	OM984422
Contig25890 ^a^	620	Rhizoctonia solani Partitivirus 14	MNR	Rhizoctonia fumigata partitivirus	62%	93%	0	OM984423
Contig59663 ^a^	589	Rhizoctonia solani Partitivirus 15	MNR	Raphanus sativus cryptic virus 1	56%	92%	0	OM984424

Note: “^a^” indicated novel partitiviruses found in MNR.

**Table 4:** The information of 26 contigs found in binucleate *Rhizoctonia* (BNR) and 28 contigs found in multinucleate *Rhizoctonia* (MNR) related to RNA-dependent RNA polymerase of mycoviruses associated with the family *Mitoviridae*.

Contig	Size (Amino Acid)	Name	Origin	Best Match	Identity	Query Cover	E-Value	Accession Number
First_Contig100 ^a,b^	767	Binucleate Rhizoctonia mitovirus 1	BNR	Rhizoctonia solani mitovirus 15	43%	71%	0	OM984453
Contig385 ^a^	857	Binucleate Rhizoctonia mitovirus 2	BNR	Rhizoctonia solani mitovirus 78	52%	67%	0	OM984454
Contig462 ^a^	959	Binucleate Rhizoctonia mitovirus 3	BNR	Rhizoctonia solani mitovirus 51	72%	73%	0	OM984455
Contig463	966	Rhizoctonia solani mitovirus 51-BNR	BNR	Rhizoctonia solani mitovirus 51	97%	73%	0	OM984456
Contig824	946	Rhizoctonia solani mitovirus 50-BNR	BNR	Rhizoctonia solani mitovirus 50	96%	92%	0	OM984457
Contig2147 ^a^	779	Binucleate Rhizoctonia mitovirus 4	BNR	Rhizoctonia solani mitovirus 15	42%	72%	0	OM984458
First_Contig193 ^a^	788	Binucleate Rhizoctonia mitovirus 5	BNR	Grapevine-associated mitovirus 10	71%	70%	0	OM984459
Contig1318 ^a^	792	Binucleate Rhizoctonia mitovirus 6	BNR	Grapevine-associated mitovirus 10	71%	66%	0	OM984460
Contig1296	833	Rhizoctonia solani mitovirus 88-BNR	BNR	Rhizoctonia solani mitovirus 88	92%	66%	0	OM984461
First_Contig3	785	Rhizoctonia solani mitovirus 101-BNR	BNR	Rhizoctonia solani mitovirus 101	98%	77%	0	OM984462
Contig2865 ^a,b^	835	Binucleate Rhizoctonia mitovirus 7	BNR	Rhizoctonia solani mitovirus 15	47%	75%	0	OM984463
Contig109 ^a^	927	Binucleate Rhizoctonia mitovirus 8	BNR	Rhizoctonia solani mitovirus 42	83%	74%	0	OM984464
Contig377	844	Rhizoctonia cerealis mitovirus-BNR	BNR	Rhizoctonia cerealis mitovirus	93%	79%	0	OM984465
Contig2511	780	Rhizoctonia solani mitovirus 1-BNR	BNR	Rhizoctonia solani mitovirus 1	100%	92%	0	OM984466
Contig139 ^a^	900	Binucleate Rhizoctonia mitovirus 9	BNR	Rhizoctonia solani mitovirus 31	61%	74%	0	OM984467
First_Contig8 ^a,b^	903	Binucleate Rhizoctonia mitovirus 10	BNR	Rhizoctonia solani mitovirus 41	84%	74%	0	OM984468
Contig461 ^a^	795	Binucleate Rhizoctonia mitovirus 11	BNR	Rhizoctonia solani mitovirus 41	56%	73%	0	OM984469
Contig301 ^a^	811	Binucleate Rhizoctonia mitovirus 12	BNR	Rhizoctonia solani mitovirus 50	89%	93%	0	OM984470
Second_Contig95	842	Rhizoctonia solani mitovirus 40-BNR	BNR	Rhizoctonia solani mitovirus 40	97%	57%	0	OM984471
Contig643 ^a,b^	568	Binucleate Rhizoctonia mitovirus 13	BNR	Epicoccum nigrum mitovirus 1	72%	68%	0	OM984472
Contig1166 ^a^	852	Binucleate Rhizoctonia mitovirus 14	BNR	Rhizoctonia solani mitovirus 78	62%	68%	0	OM984473
Contig2240 ^a^	856	Binucleate Rhizoctonia mitovirus 15	BNR	Rhizoctonia solani mitovirus 78	55%	65%	0	OM984474
Contig333 ^a,b^	1060	Binucleate Rhizoctonia mitovirus 16	BNR	Rhizoctonia solani mitovirus 43	73%	79%	0	OM984475
Contig728 ^a^	1054	Binucleate Rhizoctonia mitovirus 17	BNR	Rhizoctonia solani mitovirus 64	52%	73%	0	OM984476
Contig2765	1047	Rhizoctonia solani mitovirus 64-BNR	BNR	Rhizoctonia solani mitovirus 64	91%	73%	0	OM984477
Contig132 a	1079	Binucleate Rhizoctonia mitovirus 18	BNR	Rhizoctonia solani mitovirus 48	53%	64%	0	OM984478
Contig7624 ^a,b^	1064	Rhizoctonia solani mitovirus 105	MNR	Rhizoctonia solani mitovirus 48	50%	67%	0	OM984425
Contig787 ^a,b^	995	Rhizoctonia solani mitovirus 106	MNR	Rhizoctonia solani mitovirus 78	54%	60%	0	OM984426
Contig722	917	Rhizoctonia solani mitovirus 13-MNR	MNR	Rhizoctonia solani mitovirus 13	91%	67%	0	OM984427
Contig944 ^a^	879	Rhizoctonia solani mitovirus 107	MNR	Rhizoctonia solani mitovirus 54	64%	70%	0	OM984428
First_Contig217 ^a^	1027	Rhizoctonia solani mitovirus 108	MNR	Rhizoctonia solani mitovirus 48	86%	83%	0	OM984429
First_Contig20	864	Rhizoctonia solani mitovirus 87-MNR	MNR	Rhizoctonia solani mitovirus 87	97%	71%	0	OM984430
Contig665 ^a^	865	Rhizoctonia solani mitovirus 109	MNR	Rhizoctonia solani mitovirus 65	61%	66%	0	OM984431
Contig931	1029	Rhizoctonia solani mitovirus 95-MNR	MNR	Rhizoctonia solani mitovirus 95	96%	54%	0	OM984432
Contig201 ^a^	858	Rhizoctonia solani mitovirus 110	MNR	Rhizoctonia solani mitovirus 15	46%	52%	9 × 10^−165^	OM984433
Contig493 ^a^	836	Rhizoctonia solani mitovirus 111	MNR	Rhizoctonia solani mitovirus 88	85%	69%	0	OM984434
First_Contig34 ^a^	862	Rhizoctonia solani mitovirus 112	MNR	Rhizoctonia solani mitovirus 65	59%	65%	0	OM984435
Contig9722 ^a^	1028	Rhizoctonia solani mitovirus 113	MNR	Macrophomina phaseolina mitovirus 3	86%	74%	0	OM984436
First_Contig4017 ^a,b^	780	Rhizoctonia solani mitovirus 114	MNR	Rhizoctonia solani mitovirus 73	50%	51%	0	OM984437
First_Contig23	782	Rhizoctonia solani mitovirus 75-MNR	MNR	Rhizoctonia solani mitovirus 75	96%	60%	0	OM984438
Contig451	961	Rhizoctonia solani mitovirus 15-MNR	MNR	Rhizoctonia solani mitovirus 15	99%	73%	0	OM984439
Contig999 ^a^	939	Rhizoctonia solani mitovirus 115	MNR	Clitocybe odora virus	39%	69%	7 × 10^−160^	OM984440
Contig1235	810	Rhizoctonia cerealis mitovirus-MNR	MNR	Rhizoctonia cerealis mitovirus	92%	81%	0	OM984441
Contig3325 ^b^	855	Rhizoctonia solani mitovirus 63-MNR	MNR	Rhizoctonia solani mitovirus 63	90%	78%	0	OM984442
Contig2267 ^a^	841	Rhizoctonia solani mitovirus 116	MNR	Rhizoctonia solani mitovirus 31	45%	49%	2 × 10^−175^	OM984443
Contig1401 ^a^	900	Rhizoctonia solani mitovirus 117	MNR	Rhizoctonia solani mitovirus 31	60%	74%	0	OM984444
First_Contig722 ^a^	845	Rhizoctonia solani mitovirus 118	MNR	Rhizoctonia solani mitovirus 60	81%	75%	0	OM984445
Contig424 ^a^	843	Rhizoctonia solani mitovirus 119	MNR	Rhizoctonia solani mitovirus 78	80%	74%	0	OM984446
Contig2296 ^a^	964	Rhizoctonia solani mitovirus 120	MNR	Rhizoctonia solani mitovirus 93	64%	46%	0	OM984447
Contig5349 ^a^	882	Rhizoctonia solani mitovirus 121	MNR	Rhizoctonia solani mitovirus 84	88%	72%	0	OM984448
Second_Contig262 ^a^	421	Rhizoctonia solani mitovirus 122	MNR	Rhizoctonia solani mitovirus 43	74%	68%	0	OM984449
Contig1260 ^a^	1000	Rhizoctonia solani mitovirus 123	MNR	Rhizoctonia solani mitovirus 64	61%	95%	0	OM984450
First_Contig63	624	Rhizoctonia solani mitovirus 64-MNR	MNR	Rhizoctonia solani mitovirus 64	91%	91%	0	OM984451
Contig2497 ^a,b^	648	Rhizoctonia solani mitovirus 124	MNR	Rhizoctonia solani mitovirus 43	69%	77%	0	OM984452

Note: “^a^” indicated novel mitoviruses found in BNR or MNR; “^b^” indicated mycoviruses selected for genome organization and multiple alignment analyses.

**Table 5:** The information of one contig found in binucleate *Rhizoctonia* (BNR) and three contigs found in multinucleate *Rhizoctonia* (MNR) related to polyprotein of mycoviruses associated with the family *Hypoviridae*.

Contig	Size (Amino Acid)	Name	Origin	Best Match	Identity	Query Cover	E-Value	Accession Number
Contig11643 ^a^	2650	Binucleate Rhizoctonia hypovirus 1	BNR	Sclerotium rolfsii hypovirus 8	37%	67%	0	OM984479
Second_Contig451 ^a^	1776	Rhizoctonia solani hypovirus 9	MNR	Lentinula edodes hypovirus 1	31%	62%	1 × 10^−168^	OM984480
First_Contig678 ^a^	3611	Rhizoctonia solani hypovirus 10	MNR	Rhizoctonia solani hypovirus 1	50%	81%	0	OM984481
Contig2151 ^a^	2974	Rhizoctonia solani hypovirus 11	MNR	Mycosphaerella hypovirus A	30%	59%	7 × 10^−117^	OM984482

Note: “^a^” indicated novel hypoviruses found in BNR or MNR.

## Data Availability

The sequences reported in the present manuscript have been deposited in the GenBank database under accession numbers OM984418, OM984419, OM984420, OM984421, OM984422, OM984423, OM984424, OM984453, OM984454, OM984455, OM984456, OM984457, OM984458, OM984459, OM984460, OM984461, OM984462, OM984463, OM984464, OM984465, OM984466, OM984467, OM984468, OM984469, OM984470, OM984471, OM984472, OM984473, OM984474, OM984475, OM984476, OM984477, OM984478, OM984425, OM984426, OM984427, OM984428, OM984429, OM984430, OM984431, OM984432, OM984433, OM984434, OM984435, OM984436, OM984437, OM984438, OM984439, OM984440, OM984441, OM984442, OM984443, OM984444, OM984445, OM984446, OM984447, OM984448, OM984449, OM984450, OM984451, OM984452, OM984479, OM984480, OM984481, and OM984482.

## Supplementary Materials

The following supporting information can be downloaded at: https://www.mdpi.com/article/10.3390/jof9020214/s1, Table S1: Information of strains of binucleate *Rhizoctonia* (BNR) and multinucleate *Rhizoctonia* (MNR) isolated from diseased potatoes with symptoms of stem canker or black scurf, [27,36,41]; Table S2: Alignment information of putative mycoviruses found in binucleate *Rhizoctonia* (BNR) and multinucleate *Rhizoctonia* (MNR) using metatranscriptome sequencing.
