# Supplementary material for: Diversity of Mycoviruses Present in Strains of Binucleate Rhizoctonia and Multinucleate Rhizoctonia, Causal Agents for Potato Stem Canker or Black Scurf
Source: J Fungi (Basel). 2023 Feb 6;9(2):214. doi: 10.3390/jof9020214 (PMC9967303; doi:10.3390/jof9020214)
Supplement: Supplementary file 1 [file jof-09-00214-s001.zip › Table S1.pdf]

Table S1. Information of strains of binucleate *Rhizoctonia* (BNR) and multinucleate *Rhizoctonia* (MNR) isolated from diseased potatoes with symptoms of stem canker or black scurf.

Strain code	Anastomosis group or subgroup	Karyotype	Sampling site	Origin
GS-27	AG-3 PT	Multinucleate	Lanzhou city, Gansu province	Reference 27
GS-23	AG-3 PT	Multinucleate	Linxia city, Gansu province	Reference 27
GS-1-1	AG-3 PT	Multinucleate	Wuwei city, Gansu province	Reference 27
GS-2	AG-3 PT	Multinucleate	Wuwei city, Gansu province	Reference 27
GS-1-2	AG-K	Binucleate	Wuwei city, Gansu province	Reference 36
GS-6H	AG-5	Multinucleate	Lanzhou city, Gansu province	Reference 27
GS-9	AG-5	Multinucleate	Dingxi city, Gansu province	Reference 27
GS-19	AG-K	Binucleate	Linxia city, Gansu province	Reference 36
GS-6	AG-K	Binucleate	Lanzhou city, Gansu province	Reference 36
GS-11	AG-K	Binucleate	Dingxi city, Gansu province	Reference 36
GS-13-1	AG-5	Multinucleate	Dingxi city, Gansu province	Reference 27
GS-8	AG-5	Multinucleate	Dingxi city, Gansu province	Reference 27
GS-24	AG-4HGII	Multinucleate	Baiyin city, Gansu province	Reference 27
GS-25	AG-4HGII	Multinucleate	Baiyin city, Gansu province	Reference 23
GS-4	AG-5	Multinucleate	Lanzhou city, Gansu province	Reference 27
GS-20	AG-5	Multinucleate	Linxia city, Gansu province	Reference 27
GS-28	AG-3 PT	Multinucleate	Dingxi city, Gansu province	Reference 27
GS-34	AG-3 PT	Multinucleate	Zhangye city, Gansu province	Reference 27
GS-38	AG-A	Binucleate	Zhangye city, Gansu province	Reference 36
GS-30H	AG-3 PT	Multinucleate	Wuwei city, Gansu province	Reference 27
GS-12	AG-3 PT	Multinucleate	Dingxi city, Gansu province	Reference 27
NX-11	AG-5	Multinucleate	Guyuan city, Ningxia Hui autonomous region	Reference 27
NX-2	AG-3 PT	Multinucleate	Guyuan city, Ningxia Hui autonomous region	Reference 27

NX-5	AG-3 PT	Multinucleate	Guyuan city, Ningxia Hui autonomous region	Reference 27
NXSG-12X	AG-3 PT	Multinucleate	Guyuan city, Ningxia Hui autonomous region	This study
NXSG-11X	AG-3 PT	Multinucleate	Guyuan city, Ningxia Hui autonomous region	This study
NXSG-3X	AG-3 PT	Multinucleate	Guyuan city, Ningxia Hui autonomous region	This study
NXSG-4X	AG-3 PT	Multinucleate	Guyuan city, Ningxia Hui autonomous region	This study
NXSG-6X	AG-3 PT	Multinucleate	Guyuan city, Ningxia Hui autonomous region	This study
NXSG-8X	AG-3 PT	Multinucleate	Guyuan city, Ningxia Hui autonomous region	This study
NXSG-9X	AG-3 PT	Multinucleate	Guyuan city, Ningxia Hui autonomous region	This study
NXZB-1X	AG-4HGII	Multinucleate	Guyuan city, Ningxia Hui autonomous region	This study
NXZB-3X	AG-4HGII	Multinucleate	Guyuan city, Ningxia Hui autonomous region	This study
NXSG-5X	AG-3 PT	Multinucleate	Guyuan city, Ningxia Hui autonomous region	This study
NXZB-2X	AG-5	Multinucleate	Guyuan city, Ningxia Hui autonomous region	This study
NXSG-2X	AG-3 PT	Multinucleate	Guyuan city, Ningxia Hui autonomous region	This study
NXSG-1X	AG-3 PT	Multinucleate	Guyuan city, Ningxia Hui autonomous region	This study
NWYS-1	AG-3 PT	Multinucleate	Wuzhong city, Ningxia Hui autonomous region	This study
NXSG-10X	AG-3 PT	Multinucleate	Guyuan city, Ningxia Hui autonomous region	This study
NWYS-2	AG-3 PT	Multinucleate	Wuzhong city, Ningxia Hui autonomous region	This study
NWYS-6	AG-3 PT	Multinucleate	Wuzhong city, Ningxia Hui autonomous region	This study
NWYS-4	AG-3 PT	Multinucleate	Wuzhong city, Ningxia Hui autonomous region	This study
HeB-13-1	AG-3 PT	Multinucleate	Chengde city, Hebei province	Reference 27
HBZJ-4X	AG-5	Multinucleate	Zhangjiakou city, Hebei province	This study
HBZJ-5X	AG-3 PT	Multinucleate	Zhangjiakou city, Hebei province	This study
HBZJ-2X	AG-4HGII	Multinucleate	Zhangjiakou city, Hebei province	This study
HeB-17	AG-5	Multinucleate	Tangshan city, Hebei province	Reference 27
HeB-18	AG-5	Multinucleate	Qinhuangdao city, Hebei province	Reference 27
HeB-20	AG-4HGII	Multinucleate	Zhangjiakou city, Hebei province	Reference 27

HBZJ-3X	AG-4HGII	Multinucleate	Zhangjiakou city, Hebei province	This study
HBZJ-1X	AG-4HGI	Multinucleate	Zhangjiakou city, Hebei province	This study
YN-17H	AG-3 PT	Multinucleate	Kunming city, Yunnan province	Reference 27
YNKY-2X	AG-A	Binucleate	Kaiyuan city, Yunnan province	This study
YNKY-4X	AG-A	Binucleate	Kaiyuan city, Yunnan province	This study
YNKY-1X	AG-A	Binucleate	Kaiyuan city, Yunnan province	This study
YNKY-5X	AG-A	Binucleate	Kaiyuan city, Yunnan province	This study
YNKY-3X	AG-A	Binucleate	Kaiyuan city, Yunnan province	This study
YN-5H	AG-3 PT	Multinucleate	Kunming city, Yunnan province	Reference 27
DDJJ-2	AG-A	Binucleate	Datong city, Shanxi province	This study
DDJJ-1	AG-A	Binucleate	Datong city, Shanxi province	This study
SHMM-7	AG-A	Binucleate	Shuozhou city, Shanxi province	This study
DDJJ-3	AG-A	Binucleate	Datong city, Shanxi province	This study
SHMM-1	AG-A	Binucleate	Shuozhou city, Shanxi province	This study
YJLC-4	AG-A	Binucleate	Yangquan city, Shanxi province	This study
XKGS-12	AG-K	Binucleate	Xinzhou city, Shanxi province	This study
YJLC-2	AG-A	Binucleate	Yangquan city, Shanxi province	This study
XKGS-10	AG-K	Binucleate	Xinzhou city, Shanxi province	This study
XKGS-13	AG-K	Binucleate	Xinzhou city, Shanxi province	This study
XKGS-9	AG-K	Binucleate	Xinzhou city, Shanxi province	This study
JSZS-3	AG-A	Binucleate	Jinzhong city, Shanxi province	This study
SHMM-5	AG-K	Binucleate	Shuozhou city, Shanxi province	This study
YJLC-3	AG-A	Binucleate	Yangquan city, Shanxi province	This study
XKGS-8	AG-K	Binucleate	Xinzhou city, Shanxi province	This study
YJLC-1	AG-A	Binucleate	Yangquan city, Shanxi province	This study
SHMM-2	AG-A	Binucleate	Shuozhou city, Shanxi province	This study

SHMM-3	AG-A	Binucleate	Shuozhou city, Shanxi province	This study
SHMM-4	AG-A	Binucleate	Shuozhou city, Shanxi province	This study
SHMM-6	AG-A	Binucleate	Shuozhou city, Shanxi province	This study
XJDS-11	AG-5	Multinucleate	Xinzhou city, Shanxi province	This study
LLSY-12	AG-5	Multinucleate	Lvliang city, Shanxi province	This study
XKSA-8	AG-3 PT	Multinucleate	Xinzhou city, Shanxi province	This study
XJDM-2	AG-3 PT	Multinucleate	Xinzhou city, Shanxi province	This study
XKGS-14	AG-3 PT	Multinucleate	Xinzhou city, Shanxi province	This study
YJLC-5	AG-A	Binucleate	Yangquan city, Shanxi province	This study
XJDS-10	AG-3 PT	Multinucleate	Xinzhou city, Shanxi province	This study
XKGS-5	AG-3 PT	Multinucleate	Xinzhou city, Shanxi province	This study
XJDM-5	AG-3 PT	Multinucleate	Xinzhou city, Shanxi province	This study
XKLQ-1	AG-3 PT	Multinucleate	Xinzhou city, Shanxi province	This study
XJDS-3	AG-3 PT	Multinucleate	Xinzhou city, Shanxi province	This study
XJDS-8	AG-3 PT	Multinucleate	Xinzhou city, Shanxi province	This study
XKSA-16	AG-3 PT	Multinucleate	Xinzhou city, Shanxi province	This study
XJDS-12	AG-5	Multinucleate	Xinzhou city, Shanxi province	This study
XJDS-5	AG-3 PT	Multinucleate	Xinzhou city, Shanxi province	This study
DHXM-8	AG-3 PT	Multinucleate	Datong city, Shanxi province	This study
XKSA-12	AG-3 PT	Multinucleate	Xinzhou city, Shanxi province	This study
DHXM-16	AG-3 PT	Multinucleate	Datong city, Shanxi province	This study
XKSA-10	AG-3 PT	Multinucleate	Xinzhou city, Shanxi province	This study
SCCH-4	AG-3 PT	Multinucleate	Changzhi city, Shanxi province	This study
XJDM-3	AG-3 PT	Multinucleate	Xinzhou city, Shanxi province	This study
XKSA-11	AG-3 PT	Multinucleate	Xinzhou city, Shanxi province	This study
XKSA-9	AG-3 PT	Multinucleate	Xinzhou city, Shanxi province	This study

AH-2	AG-Fa	Binucleate	Bengbu city, Anhui province	Reference 36
AH-8H	AG-3 PT	Multinucleate	Chuzhou city, Anhui province	Reference 27
AH-4	AG-A	Binucleate	Chuzhou city, Anhui province	Reference 36
AH-1	AG-4HGI	Multinucleate	Bengbu city, Anhui province	Reference 27
AH-10H	AG-3 PT	Multinucleate	Chuzhou city, Anhui province	Reference 27
AH-2H	AG-3 PT	Multinucleate	Chuzhou city, Anhui province	Reference 27
AH-20H	AG-3 PT	Multinucleate	Ma'anshan city, Anhui province	Reference 27
AH-21H	AG-3 PT	Multinucleate	Ma'anshan city, Anhui province	Reference 27
LN-5	AG-A	Binucleate	Fuxin city, Liaoning province	Reference 36
LN-6	AG-A	Binucleate	Dandong city, Liaoning province	Reference 36
LN-2	AG-K	Binucleate	Fuxin city, Liaoning province	Reference 36
LN-4	AG-K	Binucleate	Fuxin city, Liaoning province	Reference 36
LN-7	AG-A	Binucleate	Dandong city, Liaoning province	Reference 36
LN-4H	AG-3 PT	Multinucleate	Shenyang city, Liaoning province	Reference 27
LN-1H	AG-3 PT	Multinucleate	Shenyang city, Liaoning province	Reference 27
LN-6H	AG-3 PT	Multinucleate	Shenyang city, Liaoning province	Reference 27
SC-8	AG-A	Binucleate	Nanchong city, Sichuan province	Reference 36
SC-7	AG-A	Binucleate	Dazhou city, Sichuan province	Reference 36
SC-9	AG-5	Multinucleate	Nanchong city, Sichuan province	Reference 27
SC-1	AG-5	Multinucleate	Guang'an city, Sichuan province	Reference 27
SC-9H	AG-3 PT	Multinucleate	Suining city, Sichuan province	Reference 27
SC-2	AG-A	Binucleate	Guang'an city, Sichuan province	Reference 36
SC-6	AG-A	Binucleate	Dazhou city, Sichuan province	Reference 36
SC-3	AG-A	Binucleate	Dazhou city, Sichuan province	Reference 36
SC-5	AG-4HGI	Multinucleate	Dazhou city, Sichuan province	Reference 27
HL-47	AG-A	Binucleate	Qiqihar city, Heilongjiang province	Reference 36

HL-51	AG-A	Binucleate	Qiqihar city, Heilongjiang province	Reference 36
HL-20-1	AG-A	Binucleate	Daqing city, Heilongjiang province	Reference 36
CSF	AG-4HGI	Multinucleate	Qiqihar city, Heilongjiang province	Reference 27
QYD	AG-4HGI	Multinucleate	Qiqihar city, Heilongjiang province	Reference 27
HL-15	AG-4HGIII	Multinucleate	Qiqihar city, Heilongjiang province	Reference 27
HL-12	AG-4HGIII	Multinucleate	Qiqihar city, Heilongjiang province	Reference 27
HL-6	AG-4HGIII	Multinucleate	Qiqihar city, Heilongjiang province	Reference 27
HL-1H	AG-3 PT	Multinucleate	Qiqihar city, Heilongjiang province	Reference 27
HL-41	AG-4HGI	Multinucleate	Qiqihar city, Heilongjiang province	Reference 27
HL-ZA	AG-W	Binucleate	Qiqihar city, Heilongjiang province	Reference 41
HL-39	AG-A	Binucleate	Heihe city, Heilongjiang province	Reference 36
HL-CZ	AG-W	Binucleate	Qiqihar city, Heilongjiang province	Reference 41
HL-21	AG-4HGII	Multinucleate	Qiqihar city, Heilongjiang province	Reference 27
HL-4-2	AG-4HGI	Multinucleate	Qiqihar city, Heilongjiang province	Reference 27
HL-2-1	AG-4HGIII	Multinucleate	Qiqihar city, Heilongjiang province	Reference 27
HL-46	AG-5	Multinucleate	Qiqihar city, Heilongjiang province	Reference 27
HL-5	AG-5	Multinucleate	Qiqihar city, Heilongjiang province	Reference 27
HL-1	AG-4HGIII	Multinucleate	Qiqihar city, Heilongjiang province	Reference 27
HL-36	AG-5	Multinucleate	Greater Khingan administrative office, Heilongjiang province	Reference 27
HL-19	AG-4HGI	Multinucleate	Daqing city, Heilongjiang province	Reference 27
HL-16	AG-5	Multinucleate	Qiqihar city, Heilongjiang province	Reference 27
HL-8	AG-4HGI	Multinucleate	Qiqihar city, Heilongjiang province	Reference 27
HL-45	AG-4HGI	Multinucleate	Qiqihar city, Heilongjiang province	Reference 27
HeN-8H	AG-3 PT	Multinucleate	Luoyang city, Henan province	Reference 27
HeN-7H	AG-3 PT	Multinucleate	Luoyang city, Henan province	Reference 27
HeN-10H	AG-3 PT	Multinucleate	Luoyang city, Henan province	Reference 27

HeN-20	AG-4HGI	Multinucleate	Zhoukou city, Henan province	Reference 27
ZJ-2-2	AG-A	Binucleate	Rui'an city, Zhejiang province	Reference 36
ZJ-4-2	AG-A	Binucleate	Rui'an city, Zhejiang province	Reference 36
ZJJH-3X	AG-A	Binucleate	Jinhua city, Zhejiang province	This study
ZJJH-1X	AG-A	Binucleate	Jinhua city, Zhejiang province	This study
ZJJH-2X	AG-A	Binucleate	Jinhua city, Zhejiang province	This study
ZJJH-6X	AG-A	Binucleate	Jinhua city, Zhejiang province	This study
ZJJH-5X	AG-A	Binucleate	Jinhua city, Zhejiang province	This study
ZJ-4-1	AG-A	Binucleate	Rui'an city, Zhejiang province	Reference 36
ZJJH-4X	AG-3 PT	Multinucleate	Jinhua city, Zhejiang province	This study
ZJJH-7X	AG-A	Binucleate	Jinhua city, Zhejiang province	This study
ZJ-3-1	AG-4HGI	Multinucleate	Rui'an city, Zhejiang province	Reference 27
ZJ-2-1	AG-4HGI	Multinucleate	Rui'an city, Zhejiang province	Reference 27
ZJ-5H	AG-3 PT	Multinucleate	Wenzhou city, Zhejiang province	Reference 27
SX-2	AG-Fa	Binucleate	Hanzhong city, Shaanxi province	Reference 36
SX-7	AG-K	Binucleate	Weinan city, Shaanxi province	Reference 36
SX-5	AG-5	Multinucleate	Hanzhong city, Shaanxi province	Reference 27
SX-9	AG-5	Multinucleate	Weinan city, Shaanxi province	Reference 27
BJ-1	AG-A	Binucleate	Yanqing district, Beijing municipality	Reference 36
BJYQ-1X	AG-4HGI	Multinucleate	Yanqing district, Beijing municipality	This study
BJYQ-2X	AG-4HGI	Multinucleate	Yanqing district, Beijing municipality	This study
BJYQ-12X	AG-4HGI	Multinucleate	Yanqing district, Beijing municipality	This study
BJYQ-3X	AG-4HGI	Multinucleate	Yanqing district, Beijing municipality	This study
BJYQ-4X	AG-4HGI	Multinucleate	Yanqing district, Beijing municipality	This study
BJYQ-5X	AG-4HGI	Multinucleate	Yanqing district, Beijing municipality	This study
BJYQ-6X	AG-4HGI	Multinucleate	Yanqing district, Beijing municipality	This study

BJYQ-7X	AG-4HGI	Multinucleate	Yanqing district, Beijing municipality	This study
BJYQ-8X	AG-4HGI	Multinucleate	Yanqing district, Beijing municipality	This study
BJYQ-9X	AG-4HGI	Multinucleate	Yanqing district, Beijing municipality	This study
BJYQ-10X	AG-4HGI	Multinucleate	Yanqing district, Beijing municipality	This study
BJYQ-11X	AG-4HGI	Multinucleate	Yanqing district, Beijing municipality	This study
BJYQ-13X	AG-4HGI	Multinucleate	Yanqing district, Beijing municipality	This study
BJYQ-14X	AG-4HGI	Multinucleate	Yanqing district, Beijing municipality	This study
BJYQ-15X	AG-4HGI	Multinucleate	Yanqing district, Beijing municipality	This study
BJYQ-16X	AG-4HGI	Multinucleate	Yanqing district, Beijing municipality	This study
BJYQ-17X	AG-4HGI	Multinucleate	Yanqing district, Beijing municipality	This study
BJYQ-18X	AG-4HGI	Multinucleate	Yanqing district, Beijing municipality	This study
JL-5-1	AG-A	Binucleate	Changchun city, Jilin province	Reference 36
JL-2	AG-K	Binucleate	Changchun city, Jilin province	Reference 36
JSZJ-1X	AG-1-IB	Multinucleate	Zhenjiang city, Jiangsu province	This study
JSZJ-2X	AG-1-IB	Multinucleate	Zhenjiang city, Jiangsu province	This study
GZ-12	AG-A	Binucleate	Tongren city, Guizhou province	Reference 36
GZ-3	AG-5	Multinucleate	Liupanshui city, Guizhou province	Reference 27
GZ-17	AG-5	Multinucleate	Bijie city, Guizhou province	Reference 27
GZ-5	AG-5	Multinucleate	Qianxinan Buyi and Miao autonomous prefecture, Guizhou province	Reference 27
GZ-10	AG-A	Binucleate	Tongren city, Guizhou province	Reference 36
GZ-6	AG-4HGI	Multinucleate	Qianxinan Buyi and Miao autonomous prefecture, Guizhou province	Reference 27
GZ-20	AG-5	Multinucleate	Bijie city, Guizhou province	Reference 27
QHXN-5X	AG-3 PT	Multinucleate	Xining city, Qinghai province	This study
QHXN-9X	AG-3 PT	Multinucleate	Xining city, Qinghai province	This study
QHXN-14X	AG-3 PT	Multinucleate	Xining city, Qinghai province	This study
QHXN-19X	AG-3 PT	Multinucleate	Xining city, Qinghai province	This study

QHXN-16X	AG-5	Multinucleate	Xining city, Qinghai province	This study
QHXN-31X	AG-3 PT	Multinucleate	Xining city, Qinghai province	This study
QHXN-3X	AG-5	Multinucleate	Xining city, Qinghai province	This study
QHXN-28X	AG-3 PT	Multinucleate	Xining city, Qinghai province	This study
QHXN-2X	AG-3 PT	Multinucleate	Xining city, Qinghai province	This study
QHXN-13X	AG-3 PT	Multinucleate	Xining city, Qinghai province	This study
QHXN-10X	AG-3 PT	Multinucleate	Xining city, Qinghai province	This study
QHXN-11X	AG-3 PT	Multinucleate	Xining city, Qinghai province	This study
QHXN-1X	AG-3 PT	Multinucleate	Xining city, Qinghai province	This study
QHXN-22X	AG-3 PT	Multinucleate	Xining city, Qinghai province	This study
QHXN-26X	AG-3 PT	Multinucleate	Xining city, Qinghai province	This study
QHXN-23X	AG-3 PT	Multinucleate	Xining city, Qinghai province	This study
QHXN-30X	AG-3 PT	Multinucleate	Xining city, Qinghai province	This study
QHXN-17X	AG-3 PT	Multinucleate	Xining city, Qinghai province	This study
QHXN-15X	AG-3 PT	Multinucleate	Xining city, Qinghai province	This study
QHXN-8X	AG-3 PT	Multinucleate	Xining city, Qinghai province	This study
QHXN-25X	AG-3 PT	Multinucleate	Xining city, Qinghai province	This study
QHXN-21X	AG-3 PT	Multinucleate	Xining city, Qinghai province	This study
QHXN-4X	AG-3 PT	Multinucleate	Xining city, Qinghai province	This study
QHXN-29X	AG-3 PT	Multinucleate	Xining city, Qinghai province	This study
QHXN-27X	AG-3 PT	Multinucleate	Xining city, Qinghai province	This study
QHXN-12X	AG-3 PT	Multinucleate	Xining city, Qinghai province	This study
QHXN-6X	AG-3 PT	Multinucleate	Xining city, Qinghai province	This study
QHXN-7X	AG-3 PT	Multinucleate	Xining city, Qinghai province	This study
FJ-9H	AG-3 PT	Multinucleate	Ningde city, Fujian province	Reference 27
FJ-8H	AG-3 PT	Multinucleate	Ningde city, Fujian province	Reference 27

FJ-3H	AG-3 PT	Multinucleate	Ningde city, Fujian province	Reference 27
NM-8	AG-3 PT	Multinucleate	Ulanqab city, Inner Mongolia autonomous region	Reference 27
NM-9	AG-3 PT	Multinucleate	Ulanqab city, Inner Mongolia autonomous region	Reference 27
NM-29	AG-3 PT	Multinucleate	Ulanqab city, Inner Mongolia autonomous region	Reference 27
NM-47	AG-3 PT	Multinucleate	Ulanqab city, Inner Mongolia autonomous region	Reference 27
NM-12	AG-3 PT	Multinucleate	Ulanqab city, Inner Mongolia autonomous region	Reference 27
NM-48	AG-5	Multinucleate	Ulanqab city, Inner Mongolia autonomous region	Reference 27
NM-32	AG-K	Binucleate	Xilin Gol league, Inner Mongolia autonomous region	Reference 36
NM-9-2	AG-3 PT	Multinucleate	Ulanqab city, Inner Mongolia autonomous region	Reference 27
NM-39H	AG-3 PT	Multinucleate	Ulanqab city, Inner Mongolia autonomous region	Reference 27
NM-36H	AG-3 PT	Multinucleate	Ulanqab city, Inner Mongolia autonomous region	Reference 27
NM-18-2	AG-5	Multinucleate	Ulanqab city, Inner Mongolia autonomous region	Reference 27
NM-45	AG-5	Multinucleate	Ulanqab city, Inner Mongolia autonomous region	Reference 27
NM-20-2	AG-5	Multinucleate	Ulanqab city, Inner Mongolia autonomous region	Reference 27
NM-22-2	AG-5	Multinucleate	Ulanqab city, Inner Mongolia autonomous region	Reference 27
XJ-1H	AG-4HGI	Multinucleate	Changji Prefecture Xinjiang Uygur autonomous region	Reference 27
XJ-7	AG-5	Multinucleate	Yili Kazak autonomous prefecture, Xinjiang Uygur autonomous region	Reference 27
XJ-6	AG-5	Multinucleate	Changji prefecture Xinjiang Uygur autonomous region	Reference 27
XJ-4	AG-5	Multinucleate	Changji prefecture Xinjiang Uygur autonomous region	Reference 27
XJ-8	AG-2-1	Multinucleate	Yili Kazak autonomous prefecture, Xinjiang Uygur autonomous region	Reference 27
XJZS-4X	AG-3 PT	Multinucleate	Yili Kazak autonomous prefecture, Xinjiang Uygur autonomous region	This study
XJZS-1X	AG-3 PT	Multinucleate	Yili Kazak autonomous prefecture, Xinjiang Uygur autonomous region	This study
CQ-14H	AG-3 PT	Multinucleate	Xiushan county, Chongqing municipality	Reference 27
CQ-4H	AG-3 PT	Multinucleate	Pengshui county, Chongqing municipality	Reference 27
GX-4H	AG-3 PT	Multinucleate	Nanning city, Guangxi Zhuang autonomous region	Reference 27
GD-2H	AG-3 PT	Multinucleate	Yangjiang city, Guangdong province	Reference 27

HuB-7H	AG-3 PT	Multinucleate	Jingzhou city, Hubei province	Reference 27
