# Supplementary material for: Diversity of Mycoviruses Present in Strains of Binucleate Rhizoctonia and Multinucleate Rhizoctonia, Causal Agents for Potato Stem Canker or Black Scurf
Source: J Fungi (Basel). 2023 Feb 6;9(2):214. doi: 10.3390/jof9020214 (PMC9967303; doi:10.3390/jof9020214)
Supplement: Supplementary file 1 [file jof-09-00214-s001.zip › Table S2.pdf]

Table S2 Alignment information of putative mycoviruses found in binucleate *Rhizoctonia* (BNR) and multinucleate *Rhizoctonia* (MNR) using metatranscriptome sequencing.

No.	Contig ID	Origin	Length (base pair)	Best Match	Query Cover	Identity	E-value
1	Second_Contig119	BNR	1241	Rhizoctonia solani mitovirus 31	94%	78%	0
2	First_Contig2	BNR	2732	Macrophomina phaseolina mitovirus 3	99%	89%	0
3	First_Contig18	BNR	1087	Macrophomina phaseolina mitovirus 3	12%	96%	2e-15
4	First_Contig70	BNR	893	Rhizoctonia solani mitovirus 13	93%	42%	5e-57
5	First_Contig144	BNR	1236	Rhizoctonia solani mitovirus 31	64%	61%	1e-58
6	First_Contig154	BNR	3402	Rhizoctonia solani mitovirus 15	52%	49%	6e-164
7	First_Contig409	BNR	1162	Fomitiporia mediterranea mitovirus 1	99%	59%	4e-160
8	First_Contig1307	BNR	528	Rhizoctonia solani mitovirus 31	88%	66%	2e-54
9	First_Contig2516	BNR	860	Macrophomina phaseolina mitovirus 3	16%	98%	5e-15
10	First_Contig3737	BNR	1484	Binucleate Rhizoctonia mitovirus K1	97%	88%	0
11	First_Contig7320	BNR	824	Binucleate Rhizoctonia mitovirus K1	95%	85%	4e-161
12	Contig4103	BNR	697	Binucleate Rhizoctonia mitovirus K1	90%	95%	3e-100
13	First_Contig9072	BNR	1070	Binucleate Rhizoctonia mitovirus K1	99%	53%	1e-104
14	First_Contig8194	BNR	412	Rhizoctonia solani mitovirus 31	93%	67%	6e-54
15	Contig1	BNR	1327	Rhizoctonia solani mitovirus 31	68%	71%	6e-133
16	Contig80	BNR	1607	Rhizoctonia solani mitovirus 9	56%	72%	9e-139
17	Contig101	BNR	3400	Epicoccum nigrum mitovirus 1	70%	75%	0
18	Contig332	BNR	4062	Rhizoctonia solani mitovirus 4	42%	78%	0
19	Contig562	BNR	1882	Plasmopara viticola lesion associated mitovirus 32	73%	37%	2e-75
20	Contig563	BNR	1880	Mitovirus sp.	52%	39%	5e-65
21	Contig653	BNR	2382	Rhizoctonia solani mitovirus 38	74%	77%	0
22	Contig664	BNR	3094	Rhizoctonia solani mitovirus 15	58%	49%	2e-140
23	Contig673	BNR	2509	Rhizoctonia solani mitovirus 36	54%	46%	2e-98
24	Contig828	BNR	2860	Rhizoctonia solani mitovirus 11	66%	37%	5e-136

25	Contig937	BNR	2104	Rhizoctonia solani mitovirus 15	69%	53%	7e-151
26	Contig1176	BNR	1432	Rhizoctonia solani mitovirus 15	24%	59%	1e-37
27	Contig1270	BNR	338	Rhizoctonia solani mitovirus 22	99%	63%	2e-39
28	Contig1305	BNR	2502	Rhizoctonia solani mitovirus 25	70%	44%	2e-144
29	Contig2131	BNR	1455	Rhizoctonia solani mitovirus 22	76%	42%	5e-69
30	Contig3953	BNR	2511	Rhizoctonia solani narnavirus 2	46%	83%	0
31	Contig8730	BNR	2389	Rhizoctonia solani narnavirus 16	70%	44%	3e-146
32	Contig10104	BNR	451	Macrophomina phaseolina mitovirus 3	29%	84%	8e-16
33	Contig18554	BNR	860	Binucleate Rhizoctonia mitovirus K1	65%	41%	1e-30
34	Contig71160	BNR	228	Rhizoctonia mitovirus 1 RS058	98%	52%	1e-15
35	Contig20517	BNR	2392	Bremia lactucae associated narnavirus 3	93%	47%	0
36	Contig21707	BNR	691	Rhizoctonia oryzae-sativae mitovirus 1	78%	40%	3e-28
37	Contig54552	BNR	350	Rhizoctonia oryzae-sativae mitovirus 1	60%	39%	6e-09
38	Contig94713	BNR	267	Rhizoctonia oryzae-sativae mitovirus 1	98%	35%	1e-09
39	Contig95306	BNR	258	Rhizoctonia oryzae-sativae mitovirus 1	98%	33%	8e-08
40	Contig22408	BNR	683	Ceratobasidium mitovirus A	99%	58%	4e-64
41	Contig24558	BNR	644	Binucleate Rhizoctonia mitovirus K1	97%	85%	1e-125
42	Contig41888	BNR	870	Binucleate Rhizoctonia mitovirus K1	76%	95%	8e-148
43	Contig31480	BNR	928	Binucleate Rhizoctonia mitovirus K1	83%	85%	1e-158
44	Contig35207	BNR	274	Binucleate Rhizoctonia mitovirus K1	99%	99%	7e-54
45	Contig33496	BNR	643	Binucleate Rhizoctonia mitovirus K1	55%	45%	8e-23
46	Contig57251	BNR	212	Binucleate Rhizoctonia mitovirus K1	80%	84%	3e-24
47	Contig60390	BNR	684	Binucleate Rhizoctonia mitovirus K1	92%	98%	2e-106
48	Contig64008	BNR	347	Binucleate Rhizoctonia mitovirus K1	99%	86%	1e-62
49	Contig71154	BNR	264	Binucleate Rhizoctonia mitovirus K1	100%	93%	2e-47
50	Contig87001	BNR	292	Binucleate Rhizoctonia mitovirus K1	99%	94%	5e-55

51	Contig90891	BNR	458	Binucleate Rhizoctonia mitovirus K1	58%	78%	1e-35
52	Contig106434	BNR	228	Binucleate Rhizoctonia mitovirus K1	86%	47%	3e-09
53	Contig6291	BNR	2513	Plasmopara viticola lesion associated ourmia-like virus 25	49%	38%	2e-75
54	Contig14760	BNR	2479	Apple ourmia-like virus 3	48%	42%	2e-82
55	Contig82664	BNR	598	Rhizoctonia solani ourmia-like virus 5	90%	52%	4e-44
56	Contig27340	BNR	1173	Rhizoctonia solani ourmia-like virus 5	91%	36%	4e-60
57	First_Contig332	BNR	8630	Rhizoctonia solani fusarivirus 3	50%	41%	0
58	Contig44768	BNR	434	Rhizoctonia solani fusarivirus 2	99%	49%	2e-37
59	Contig36084	BNR	493	Rhizoctonia solani fusarivirus 1	70%	45%	1e-08
60	Contig34159	BNR	410	Rhizoctonia solani fusarivirus 1	96%	39%	7e-20
61	Contig53345	BNR	780	Rhizoctonia solani fusarivirus 2	99%	39%	6e-50
62	Contig62229	BNR	620	Rhizoctonia solani fusarivirus 1	57%	38%	2e-15
63	Contig68539	BNR	341	Rhizoctonia solani fusarivirus 1	98%	50%	3e-24
64	Contig74796	BNR	345	Rhizoctonia solani fusarivirus 1	100%	65%	5e-45
65	Contig85663	BNR	359	Rhizoctonia solani fusarivirus 2	98%	57%	1e-39
66	Contig89488	BNR	323	Rhizoctonia solani fusarivirus 1	99%	89%	2e-59
67	Contig95547	BNR	284	Rhizoctonia solani fusarivirus 2	97%	63%	3e-33
68	Contig96886	BNR	366	Rhizoctonia solani fusarivirus 2	86%	39%	6e-17
69	First_Contig9011 [a]	BNR	781	Soybean leaf-associated negative-stranded RNA virus 4	99%	37%	2e-38
70	Contig45942	BNR	673	Soybean leaf-associated negative-stranded RNA virus 4	95%	27%	2e-15
71	Contig56190	BNR	690	Soybean leaf-associated negative-stranded RNA virus 4	88%	37%	3e-25
72	Contig61515	BNR	599	Soybean leaf-associated negative-stranded RNA virus 4	88%	28%	5e-14
73	Contig88823	BNR	443	Soybean leaf-associated negative-stranded RNA virus 4	97%	30%	2e-08
74	Contig8027	BNR	1600	Rhizoctonia solani beny-like virus 1	99%	50%	3e-175
75	Contig15540	BNR	1775	Rhizoctonia solani dsRNA virus 1	76%	67%	0
76	Contig14033	BNR	3386	Cyathus narnavirus A	42%	35%	4e-59

77	Contig110643	BNR	296	Agaricus bisporus virus 15	69%	45%	6e-11
78	Contig26831	BNR	838	Rhizoctonia solani beny-like virus 4	87%	60%	5e-100
79	Contig29937	BNR	1424	Rhizoctonia solani beny-like virus 1	71%	42%	1e-68
80	Contig32832	BNR	710	Rhizoctonia solani beny-like virus 1	99%	58%	1e-80
81	Contig47107	BNR	1431	Rhizoctonia solani ourmia-like virus 5	66%	45%	2e-59
82	Contig62035	BNR	2945	Rhizoctonia solani hypovirus 3	37%	29%	5e-27
83	Contig66387	BNR	1934	Rhizoctonia solani beny-like virus 1	43%	43%	5e-63
84	Contig40969	BNR	1684	Rhizoctonia solani dsRNA virus 1	99%	80%	0
85	First_Contig9432	BNR	2357	Trichoderma hypovirus	43%	32%	6e-40
86	Contig50802	BNR	1152	Sclerotium rolfsii hypovirus 4	96%	32%	4e-43
87	Contig73093	BNR	787	Ceratobasidium hypovirus-like	95%	30%	4e-23
88	First_Contig10271	BNR	383	Sclerotinia sclerotiorum negative-stranded RNA virus 4	83%	41%	3e-13
89	Contig82126	BNR	670	Rhizoctonia solani endornavirus-RS002	99%	96%	2e-149
90	First_Contig11029	BNR	645	Rhizoctonia solani endornavirus-RS058-1	53%	96%	1e-73
91	Contig50008	BNR	380	Rhizoctonia solani endornavirus-RS058-1	99%	96%	3e-82
92	Contig82339	BNR	270	Rhizoctonia solani endornavirus-RS006-2	98%	99%	8e-53
93	Contig109202	BNR	251	Rhizoctonia solani endornavirus-RS006-2	99%	96%	1e-46
94	Contig89133	BNR	325	Rhizoctonia solani endornavirus-RS024-1	72%	100%	5e-47
95	Contig91391	BNR	311	Rhizoctonia solani endornavirus-RS058-1	99%	100%	7e-68
96	Contig91548	BNR	282	Rhizoctonia solani endornavirus-RS058-1	98%	92%	4e-54
97	Contig10662	BNR	329	Morchella importuna endornavirus 1	99%	51%	6e-28
98	Contig12120	BNR	1064	Ceratobasidium endornavirus E	95%	34%	9e-33
99	Contig15704	BNR	1762	Ceratobasidium endornavirus E	95%	38%	2e-111
100	Contig49209	BNR	440	Ceratobasidium endornavirus E	86%	38%	3e-24
101	Contig69335	BNR	311	Ceratobasidium endornavirus F	98%	53%	2e-22
102	Contig72702	BNR	356	Ceratobasidium endornavirus F	98%	49%	4e-28

103	Contig91043	BNR	216	Ceratobasidium endornavirus A	90%	71%	4e-23
104	Contig92293	BNR	570	Ceratobasidium endornavirus F	98%	32%	3e-17
105	Contig99476	BNR	273	Rhizoctonia solani endornavirus 5	98%	51%	2e-23
106	Contig111073	BNR	363	Rhizoctonia solani endornavirus 4	86%	35%	6e-12
107	Contig114961	BNR	305	Rhizoctonia solani endornavirus 4	99%	38%	2e-12
108	Contig114823	BNR	396	Morchella importuna endornavirus 1	92%	39%	2e-21
109	Contig116720	BNR	219	Morchella importuna endornavirus 2	95%	45%	1e-10
110	Contig83395	BNR	309	Rhizoctonia solani endornavirus 6	100%	68%	4e-39
111	Contig80571	BNR	784	Rosellinia necatrix partitivirus 5	71%	34%	2e-18
112	Contig91437	BNR	404	Rosellinia necatrix partitivirus 5	78%	34%	3e-12
113	Contig106957	BNR	316	Rhizoctonia solani partitivirus 6	95%	75%	1e-40
114	Contig37038	BNR	1904	Trichoderma atroviride partitivirus 1	96%	59%	0
115	Contig67140	BNR	427	Rhizoctonia solani dsRNA virus 1	65%	62%	4e-35
116	Contig72348	BNR	288	Rhizoctonia solani dsRNA virus 1	63%	70%	5e-19
117	Contig115536	BNR	296	Rhizoctonia solani dsRNA virus 2	99%	100%	5e-62
118	Contig47505	BNR	796	Rhizoctonia solani partitivirus 6	89%	72%	2e-117
119	Contig104035	BNR	208	Heterobasidion partitivirus 15	93%	77%	2e-26
120	Contig114545	BNR	306	Heterobasidion RNA virus 6	99%	63%	8e-35
121	Contig34240	BNR	455	Sclerotium rolfsii unassigned dsRNA virus 2	98%	54%	4e-48
122	Contig72814	BNR	259	Sclerotium rolfsii unassigned dsRNA virus 2	98%	53%	3e-18
123	Contig85037	BNR	768	Rhizoctonia fumigata mycovirus	90%	70%	5e-91
124	Contig104756	BNR	375	Rhizoctonia fumigata mycovirus	99%	36%	3e-09
125	Contig112162	BNR	411	Rhizoctonia fumigata mycovirus	98%	72%	6e-62
126	Contig87186	BNR	585	Beihai razor shell virus 4	42%	37%	3e-07
127	Contig92291	BNR	270	Sclerotium rolfsii unassigned dsRNA virus 2	96%	66%	8e-23
128	Contig93617	BNR	357	Colletotrichum truncatum partitivirus 1	84%	80%	1e-50

129	Contig60011	BNR	581	Rhizoctonia solani beny-like virus 1	97%	46%	7e-29
130	Contig63614	BNR	632	Rhizoctonia solani beny-like virus 1	98%	45%	3e-57
131	Contig66574	BNR	598	Rhizoctonia solani beny-like virus 1	60%	53%	5e-34
132	Contig71291	BNR	268	Rhizoctonia solani beny-like virus 1	98%	68%	6e-36
133	Contig83110	BNR	677	Rhizoctonia solani beny-like virus 1	99%	67%	4e-61
134	Contig88989	BNR	635	Rhizoctonia solani beny-like virus 1	98%	61%	6e-82
135	Contig89817	BNR	362	Rhizoctonia solani beny-like virus 1	96%	55%	3e-34
136	Contig92007	BNR	827	Rhizoctonia solani beny-like virus 1	94%	56%	4e-106
137	Contig38246	BNR	606	Erysiphe necator associated ourmia-like virus 100	99%	64%	7e-82
138	Contig75407 [a]	BNR	315	Dougjudy virga-like virus	90%	40%	1e-15
139	Contig26437	BNR	1974	Rhizoctonia solani beny-like virus 1	92%	50%	0
140	Contig46362 [a]	BNR	1062	Soybean leaf-associated negative-stranded RNA virus 4	61%	33%	2e-23
141	Contig75184	BNR	910	Sclerotinia sclerotiorum fusarivirus 1	99%	46%	6e-82
142	Contig92899	BNR	588	Beihai sesarmid crab virus 7	95%	37%	7e-25
143	Contig100064	BNR	391	Phytophthora condilina RNA virus 2	95%	35%	6e-19
144	Contig105922	BNR	287	Rhizoctonia solani flexivirus 2	95%	54%	5e-27
145	Contig160	MNR	3744	Rhizoctonia solani mitovirus 48	34%	87%	2e-77
146	First_Contig131	MNR	3683	Rhizoctonia solani mitovirus 15	71%	75%	0
147	Contig866	MNR	3570	Epicoccum nigrum mitovirus 1	57%	55%	0
148	First_Contig106	MNR	3366	Soybean leaf-associated mitovirus 5	51%	42%	4e-126
149	First_Contig7891	MNR	3344	Rhizoctonia solani mitovirus 25	72%	46%	0
150	First_Contig31	MNR	3329	Rhizoctonia solani mitovirus 32	71%	70%	0
151	First_Contig216	MNR	3244	Rhizoctonia solani mitovirus 13	84%	55%	0
152	Contig5551	MNR	3231	Rhizoctonia solani mitovirus 11	79%	50%	0
153	Contig786	MNR	3218	Alternaria alternata mitovirus 1	78%	76%	0
154	First_Contig644	MNR	1533	Rhizoctonia solani endornavirus 1	46%	49%	2e-63

155	First_Contig682	MNR	3193	Rhizoctonia solani mitovirus 31	84%	71%	0
156	Contig1522	MNR	3184	Rhizoctonia solani mitovirus 15	73%	42%	0
157	First_Contig1213	MNR	2015	Erysiphales narna-like virus 3	53%	36%	2e-51
158	Contig1750	MNR	3121	Epicoccum nigrum mitovirus 1	77%	45%	0
159	First_Contig37	MNR	3119	Rhizoctonia solani mitovirus 1	49%	74%	0
160	Contig686	MNR	3101	Rhizoctonia solani mitovirus 15	80%	47%	0
161	Contig8319	MNR	3098	Rhizoctonia solani mitovirus 8	75%	46%	0
162	First_Contig9140	MNR	917	Ceratobasidium endornavirus C	84%	32%	1e-25
163	First_Contig9844	MNR	633	Apple ourmia-like virus 3	92%	49%	9e-51
164	First_Contig10546	MNR	614	Rhizoctonia solani endornavirus RS058-1	96%	98%	8e-130
165	First_Contig10837	MNR	343	Rhizoctonia solani fusarivirus 3	96%	76%	2e-36
166	First_Contig11717	MNR	419	Rhizoctonia solani fusarivirus 1	98%	67%	2e-59
167	First_Contig11798	MNR	754	Rhizoctonia solani beny-like virus 1	50%	39%	2e-17
168	First_Contig72	MNR	3028	Rhizoctonia solani mitovirus 31	87%	79%	0
169	First_Contig50	MNR	2930	Rhizoctonia solani mitovirus 31	86%	78%	0
170	Contig2627	MNR	2878	Rhizoctonia solani mitovirus 78	69%	56%	0
171	Contig224	MNR	2823	Rhizoctonia solani mitovirus 14	68%	58%	0
172	Contig2016	MNR	2791	Rhizoctonia solani mitovirus 11	67%	42%	2e-169
173	First_Contig479	MNR	2753	Rhizoctonia solani mitovirus 14	79%	61%	0
174	Contig491	MNR	2728	Macrophomina phaseolina mitovirus 3	81%	36%	1e-116
175	First_Contig1233	MNR	2725	Epicoccum nigrum mitovirus 1	84%	53%	1e-176
176	Contig4839	MNR	2718	Rhizoctonia solani mitovirus 11	77%	39%	2e-158
177	Contig321	MNR	211	Ceratobasidium partitivirus	96%	73%	6e-27
178	Contig785	MNR	2704	Rhizoctonia solani mitovirus 38	78%	78%	0
179	Contig3042	MNR	2694	Rhizoctonia solani mitovirus 15	87%	69%	0
180	Contig78	MNR	2691	Rhizoctonia solani mitovirus 76	82%	84%	0

181	Contig4210	MNR	2667	Neofusicoccum parvum mitovirus 1	78%	88%	0
182	First_Contig170	MNR	2642	Rhizoctonia mitovirus 1	97%	99%	0
183	First_Contig425	MNR	2591	Rhizoctonia solani mitovirus 15	86%	65%	0
184	First_Contig42	MNR	2590	Rhizoctonia solani mitovirus 7	98%	54%	0
185	Contig428	MNR	2438	Rhizoctonia solani mitovirus 78	79%	81%	0
186	First_Contig177	MNR	2422	Rhizoctonia solani mitovirus 1	57%	70%	0
187	First_Contig14	MNR	2393	Rhizoctonia solani mitovirus 7	42%	53%	1e-72
188	First_Contig91	MNR	2333	Rhizoctonia solani mitovirus 13	73%	61%	0
189	First_Contig88	MNR	2314	Rhizoctonia solani mitovirus 49	89%	56%	0
190	First_Contig19	MNR	2278	Macrophomina phaseolina mitovirus 3	86%	63%	3e-105
191	First_Contig70	MNR	2256	Rhizoctonia solani mitovirus 35	49%	44%	2e-111
192	First_Contig36	MNR	2169	Rhizoctonia solani mitovirus 1	56%	68%	0
193	Contig716	MNR	1168	Epicoccum nigrum mitovirus 1	87%	67%	5e-150
194	Contig1292	MNR	2153	Epicoccum nigrum mitovirus 1	75%	72%	0
195	Second_Contig831	MNR	2544	Rhizoctonia solani fusarivirus 5	39%	44%	2e-77
196	First_Contig10	MNR	1700	Rhizoctonia solani mitovirus 25	78%	62%	0
197	Contig620	MNR	2061	Rhizoctonia solani mitovirus 21	95%	61%	0
198	First_Contig86	MNR	696	Rhizoctonia solani mitovirus 25	77%	64%	3e-70
299	Contig681	MNR	1986	Grapevine-associated mitovirus 19	82%	54%	0
200	First_Contig7391	MNR	1925	Erysiphe necator associated mitovirus 8	90%	66%	0
201	First_Contig255	MNR	1406	Lichen partiti-like RNA virus 2	90%	29%	4e-49
202	First_Contig1819	MNR	1893	Rhizoctonia solani mitovirus 4	62%	42%	1e-82
203	Contig8519	MNR	1742	Grapevine-associated mitovirus 10	65%	85%	0
204	Contig426	MNR	1741	Rhizoctonia solani mitovirus 60	78%	80%	0
205	Second_Contig17	MNR	1719	Rhizoctonia solani mitovirus 31	91%	69%	0
206	First_Contig725	MNR	396	Heterobasidion partitivirus 5	81%	56%	6e-32

207	First_Contig760	MNR	785	Ceratobasidium partitivirus	99%	65%	5e-116
208	Contig2667	MNR	1715	Rhizoctonia solani mitovirus 40	70%	83%	0
209	First_Contig871	MNR	672	Cherry chlorotic rusty spot associated partitivirus	83%	44%	5e-44
210	First_Contig941	MNR	720	Rhizoctonia solani dsRNA virus 4	99%	62%	1e-101
211	First_Contig1194	MNR	671	Gaeumannomyces tritici partitivirus 2	88%	46%	4e-52
212	Contig612	MNR	1572	Rhizoctonia solani mitovirus 65	86%	52%	8e-147
213	First_Contig1276	MNR	1078	Beihai sesarmid crab virus 7	99%	30%	6e-36
214	Contig465	MNR	1465	Rhizoctonia solani mitovirus 33	57%	63%	7e-117
215	First_Contig1893	MNR	1202	Grapevine rupestris vein feathering virus	82%	25%	4e-08
216	First_Contig3984	MNR	3270	Rhizoctonia solani fusarivirus 3	90%	64%	0
217	First_Contig4140	MNR	2730	Rhizoctonia solani fusarivirus 3	5%	64%	3e-07
218	First_Contig4586	MNR	1064	Rhizoctonia solani endornavirus-RS002	99%	99%	0
219	First_Contig6724	MNR	10581	Rhizoctonia solani fusarivirus 1	43%	72%	0
220	First_Contig6982	MNR	2349	Rhizoctonia solani endornavirus 1	78%	43%	1e-143
221	Contig5799	MNR	1456	Grapevine-associated mitovirus 10	76%	89%	0
222	First_Contig8109	MNR	2134	Rhizoctonia solani fusarivirus 3	93%	47%	6e-145
223	First_Contig8125	MNR	1551	Bipolaris oryzae hypovirus 1	49%	28%	5e-11
224	First_Contig8371	MNR	1183	Sclerotium hydrophilum virus 1	87%	77%	0
225	First_Contig8445	MNR	8619	Rhizoctonia solani endornavirus 1	46%	24%	2e-72
226	First_Contig8786 [a]	MNR	1736	Soybean leaf-associated negative-stranded RNA virus 4	99%	33%	2e-79
227	First_Contig9052	MNR	1941	Rhizoctonia solani fusarivirus 1	99%	52%	0
228	First_Contig9253	MNR	1067	Sinomenium acutum tymovirus 1	93%	72%	2e-21
229	First_Contig9863	MNR	1453	Rhizoctonia solani hypovirus 1	99%	63%	0
230	First_Contig9967	MNR	1526	Rhizoctonia solani fusarivirus 3	99%	79%	0
231	First_Contig10634	MNR	1986	Rhizoctonia solani endornavirus	100%	36%	2e-146
232	First_Contig10788	MNR	1200	Ceratobasidium endornavirus C	95%	33%	5e-51

233	First_Contig12252	MNR	1155	Rhizoctonia solani endornavirus-RS002	99%	98%	0
234	First_Contig12377	MNR	885	Ceratobasidium partitivirus	99%	37%	4e-154
235	First_Contig12664	MNR	902	Armillaria mellea ourmia-like virus 1	98%	32%	6e-28
236	Contig322 [a]	MNR	1216	Ceratobasidium partitivirus	98%	62%	6e-178
237	First_Contig577	MNR	1423	Rhizoctonia solani mitovirus 64	99%	87%	0
238	Contig7906	MNR	1390	Rhizoctonia solani mitovirus 22	78%	76%	2e-143
239	First_Contig64	MNR	1387	Rhizoctonia solani mitovirus 22	66%	75%	0
240	First_Contig180	MNR	1319	Rhizoctonia solani mitovirus 88	72%	75%	4e-158
241	First_Contig160	MNR	1312	Rhizoctonia solani mitovirus 48	34%	87%	2e-77
242	Contig281	MNR	1303	Rhizoctonia solani mitovirus 76	99%	79%	0
243	Contig466	MNR	1250	Epicoccum nigrum mitovirus 1	55%	57%	2e-131
244	Contig184	MNR	1235	Rhizoctonia solani mitovirus 15	69%	36%	3e-37
245	First_Contig1282	MNR	1133	Rhizoctonia solani mitovirus 31	72%	73%	8e-134
246	Contig303	MNR	1119	Rhizoctonia solani mitovirus 5	37%	42%	7e-13
247	Contig1062	MNR	1787	Rhizoctonia solani partitivirus 11	80%	43%	7e-125
248	First_Contig94	MNR	1114	Rhizoctonia solani mitovirus 14	68%	48%	1e-60
249	First_Contig968	MNR	1066	Rhizoctonia solani mitovirus 7	57%	64%	5e-26
250	First_Contig28	MNR	967	Rhizoctonia solani mitovirus 13	72%	46%	2e-50
251	Contig1247	MNR	2262	Rhizoctonia cerealis mitovirus	74%	83%	0
252	Contig648	MNR	941	Rhizoctonia solani mitovirus 14	87%	48%	2e-67
253	Contig680	MNR	884	Rhizoctonia solani mitovirus 64	99%	88%	1e-172
254	Contig7223	MNR	832	Rhizoctonia solani mitovirus 65	98%	80%	3e-149
255	Contig269	MNR	814	Rhizoctonia solani mitovirus 48	88%	72%	1e-109
256	First_Contig572	MNR	810	Rhizoctonia solani mitovirus 65	100%	96%	0
257	First_Contig102	MNR	800	Soybean leaf-associated mitovirus 5	59%	47%	2e-34
258	Contig1903	MNR	1076	Rhizoctonia solani fusarivirus 1	99%	43%	8e-74

259	Contig13693	MNR	788	Rhizoctonia solani mitovirus 65	94%	52%	7e-52
260	Contig2123	MNR	705	Rhizoctonia solani mitovirus 25	76%	65%	2e-71
261	First_Contig651	MNR	775	Rhizoctonia solani mitovirus 38	99%	74%	2e-120
262	Contig2728	MNR	765	Rhizoctonia solani mitovirus 64	99%	93%	1e-129
263	Contig3605	MNR	756	Rhizoctonia solani mitovirus 25	100%	61%	2e-99
264	First_Contig62	MNR	748	Rhizoctonia solani mitovirus 4	93%	56%	3e-80
265	Contig3922	MNR	744	Rhizoctonia solani mitovirus 14	95%	63%	2e-64
266	Contig2651	MNR	734	Rhizoctonia solani mitovirus 48	99%	80%	1e-128
267	First_Contig2442	MNR	733	Rhizoctonia solani mitovirus 4	93%	52%	2e-64
268	Contig2656	MNR	493	Rhizoctonia solani mitovirus 25	99%	57%	2e-54
269	Contig2660	MNR	294	Epicoccum nigrum mitovirus 1	98%	79%	3e-48
270	Contig1141	MNR	723	Rhizoctonia solani mitovirus 88	99%	84%	1e-142
271	First_Contig305	MNR	673	Rhizoctonia solani mitovirus 9	99%	78%	6e-124
272	First_Contig105	MNR	658	Rhizoctonia solani mitovirus 64	98%	90%	1e-119
273	Contig3116	MNR	283	Fomitiporia mediterranea mitovirus 1	96%	77%	2e-40
274	Contig120504	MNR	640	Binucleate Rhizoctonia mitovirus K1	95%	40%	3e-35
275	Contig3535	MNR	1117	Rhizoctonia solani narnavirus 19	51%	57%	1e-66
276	Contig7189	MNR	629	Rhizoctonia solani mitovirus 31	94%	74%	4e-50
277	Contig1832	MNR	578	Rhizoctonia solani mitovirus 21	99%	46%	8e-41
278	Contig80151	MNR	573	Rhizoctonia mitovirus 1 RS058-1	98%	43%	6e-38
279	First_Contig1443	MNR	572	Rhizoctonia solani mitovirus 55	99%	89%	1e-118
280	Contig2164	MNR	567	Macrophomina phaseolina mitovirus 3	60%	46%	1e-19
281	Contig5246	MNR	332	Heterobasidion partitivirus 5	96%	55%	2e-31
282	Contig5269	MNR	1542	Rhizoctonia solani fusarivirus 2	75%	54%	5e-142
283	Contig6544	MNR	567	Rhizoctonia solani mitovirus 63	95%	53%	3e-43
284	First_Contig641	MNR	562	Rhizoctonia solani mitovirus 36	99%	56%	7e-62

285	First_Contig408	MNR	525	Rhizoctonia solani mitovirus 88	99%	82%	2e-95
286	Contig2620	MNR	489	Rhizoctonia solani mitovirus 25	82%	60%	3e-53
287	Contig6543	MNR	1552	Rhizoctonia solani mitovirus 63	61%	47%	2e-59
288	Contig3499	MNR	483	Rhizoctonia solani mitovirus 25	99%	45%	3e-33
289	Contig1152	MNR	440	Rhizoctonia solani mitovirus 13	98%	43%	1e-25
290	Contig95483	MNR	436	Binucleate Rhizoctonia mitovirus K1	99%	90%	2e-67
291	Contig660	MNR	430	Rhizoctonia solani mitovirus 14	96%	61%	1e-46
292	Contig92951	MNR	415	Binucleate Rhizoctonia mitovirus K1	99%	83%	3e-75
293	Contig1246	MNR	405	Rhizoctonia solani mitovirus 55	97%	47%	1e-29
294	Contig547	MNR	388	Rhizoctonia solani mitovirus 55	96%	98%	1e-70
295	Contig8681	MNR	532	Rhizoctonia solani partitivirus 10	99%	100%	1e-124
296	Contig9451	MNR	1760	Rhizoctonia solani dsRNA virus 3	87%	88%	0
297	Contig129856	MNR	386	Fusarium poae mitovirus 2	98%	75%	6e-49
298	Contig10054	MNR	6500	Sinomenium acutum tymovirus 1	62%	33%	2e-137
299	Contig11085	MNR	561	Rhizoctonia solani mitovirus 119	100%	94%	9e-117
300	Contig11265	MNR	331	Ceratobasidium endornavirus E	99%	45%	2e-24
301	Contig11844	MNR	844	Rhizoctonia cerealis mitovirus	89%	88%	1e-151
302	Contig12317	MNR	276	Tomato blistering mosaic virus	95%	43%	4e-12
303	Contig13593	MNR	307	Bolbocoleon piliferum toti-like virus	97%	40%	8e-15
304	Contig13669	MNR	738	Alphaendornavirus sp.	89%	45%	5e-57
305	Contig606	MNR	379	Rhizoctonia solani mitovirus 80	49%	84%	4e-24
306	Contig15082	MNR	1737	Rhizoctonia solani dsRNA virus 2	84%	100%	0
307	Contig15103	MNR	278	Rhizoctonia solani fusarivirus 4	99%	89%	1e-51
308	Contig15771	MNR	365	Rhizoctonia solani partitivirus 11	98%	96%	1e-76
309	Contig16297	MNR	1278	Rhizoctonia solani partitivirus 10	92%	95%	0
310	Contig16400	MNR	1962	Heterobasidion partitivirus 5	91%	68%	0

311	Contig17018	MNR	1727	Rhizoctonia solani partitivirus 11	87%	79%	0
312	Contig17211	MNR	408	Agaricus bisporus virus 14	75%	37%	4e-12
313	Contig17796	MNR	1206	Rhizoctonia solani ourmia-like virus 7	58%	76%	2e-113
314	Contig18226	MNR	1853	Rhizoctonia solani dsRNA virus 3	93%	96%	0
315	Contig18683	MNR	380	Rhizoctonia solani partitivirus 11	99%	92%	2e-77
316	Contig19341	MNR	464	Rhizoctonia solani endornavirus 5	96%	50%	1e-39
317	Contig19560	MNR	292	Rhizoctonia solani endornavirus 6	93%	80%	1e-42
318	Contig19933	MNR	1758	Ceratobasidium partitivirus CP-c2	71%	32%	7e-49
319	Contig20508 [a]	MNR	619	Rhizoctonia solani negative-stranded virus 7	99%	86%	5e-128
320	Contig20650	MNR	596	Rhizoctonia cerealis mitovirus	57%	48%	2e-26
321	Contig21980 [a]	MNR	434	Rhizoctonia solani negative-stranded virus 3	99%	87%	2e-78
322	Contig27958	MNR	1070	Rhizoctonia solani fusarivirus 3	97%	40%	1e-71
323	Contig29510	MNR	1939	Sclerotium hydrophilum virus 1	46%	76%	1e-148
324	Contig29690	MNR	1402	Rhizoctonia solani narnavirus 18	99%	45%	1e-119
325	Contig30496	MNR	545	Rhizoctonia solani fusarivirus 3	98%	75%	1e-75
326	Contig30497	MNR	478	Rhizoctonia solani fusarivirus 3	99%	78%	9e-69
327	Contig30656 [a]	MNR	612	Rhizoctonia solani negative-stranded virus 2	99%	70%	1e-95
328	Contig31178	MNR	936	Rhizoctonia solani endornavirus - RS058-1	99%	95%	0
329	Contig31811	MNR	1571	XiangYun toti-like virus 7	71%	29%	8e-40
330	Contig32893	MNR	6545	Erysiphe necator associated beny-like virus 1	63%	32%	6e-118
331	Contig32998	MNR	5175	Rhizoctonia solani endornavirus	93%	33%	0
332	Contig33251	MNR	3235	Sclerotium rolfsii hypovirus 7	54%	29%	1e-30
333	Contig33646	MNR	2279	Rhizoctonia solani fusarivirus 4	50%	30%	6e-43
334	Contig33717	MNR	1064	Rhizoctonia solani fusarivirus 3	99%	87%	2e-176
335	Contig34369	MNR	481	Ceratobasidium endornavirus 1	87%	44%	3e-28
336	Contig34791	MNR	448	Rhizoctonia solani endornavirus-RS002	99%	98%	5e-91

337	Contig34792	MNR	519	Rhizoctonia solani endornavirus-RS058-1	99%	98%	5e-119
338	Contig35176	MNR	413	Rhizoctonia solani fusarivirus 4	99%	99%	1e-87
339	Contig36244	MNR	262	Plasmopara viticola associated Partitivirus 9	69%	49%	3e-11
340	Contig36646	MNR	1484	Rhizoctonia solani narnavirus 4	99%	67%	0
341	Contig37102	MNR	1770	Sinomenium acutum tymovirus 1	35%	48%	7e-54
342	Contig37997	MNR	349	Beijing alphaendornavirus 3	87%	67%	2e-30
343	Contig39868	MNR	1605	Rhizoctonia solani hypovirus 10	99%	64%	0
344	Contig42081	MNR	2601	Rhizoctonia solani fusarivirus 3	98%	50%	0
345	Contig42228	MNR	319	Rhizoctonia solani dsRNA virus 19	97%	45%	2e-22
346	Contig42359	MNR	866	Rhizoctonia solani fusarivirus 4	99%	94%	0
347	Contig42360	MNR	2906	Rhizoctonia solani fusarivirus 4	99%	88%	0
348	Contig44045	MNR	539	Monilinia fructicola beny-like virus 1	96%	49%	5e-49
349	Contig45154	MNR	1829	Rhizoctonia solani fusarivirus 4	94%	77%	0
350	Contig45966	MNR	531	Rhizoctonia solani endornavirus - RS058-1	46%	48%	1e-17
351	Contig46786	MNR	244	Rhizoctonia solani beny-like virus 1	95%	69%	1e-34
352	Contig47414	MNR	351	Rhizoctonia solani endornavirus 6	94%	52%	8e-22
353	Contig47581	MNR	402	Rhizoctonia solani fusarivirus 3	100%	75%	1e-60
354	Contig48146	MNR	818	Riboviria sp.	98%	36%	4e-44
355	Contig49793	MNR	483	Rhizoctonia solani endornavirus-RS002	100%	98%	7e-101
356	Contig49822	MNR	312	Rhizoctonia oryzae-sativae partitivirus 5	99%	54%	3e-25
357	Contig49823	MNR	341	Sarcosphaera coronaria partitivirus	89%	52%	3e-23
358	Contig50229	MNR	505	Agaricus bisporus virus 13	99%	39%	3e-23
359	Contig50570	MNR	546	Rhizoctonia solani fusarivirus 3	99%	71%	6e-65
360	Contig52202	MNR	431	Rhizoctonia solani endornavirus-RS002	99%	98%	7e-88
361	Contig52535	MNR	233	Helicobasidium purpureum partitivirus	97%	86%	3e-40
362	Contig52736	MNR	472	Rhizoctonia solani endornavirus-RS006-2	99%	99%	7e-106

363	Contig52756	MNR	494	Sichuan mosquito Beny-like virus	97%	43%	1e-34
364	Contig53116	MNR	317	Rhizoctonia solani endornavirus-RS024-1	97%	98%	2e-63
365	Contig53346 [a]	MNR	655	Soybean leaf-associated negative-stranded RNA virus 4	69%	32%	3e-16
366	Contig53647	MNR	833	Rhizoctonia solani ourmia-like virus 7	64%	58%	3e-55
367	Contig54477	MNR	362	Sinomenium acutum tymovirus 1	94%	53%	4e-31
368	Contig55181	MNR	1205	Erysiphe necator associated negative-stranded RNA virus 14	49%	27%	2e-09
369	Contig55379	MNR	787	Sarcosphaera coronaria partitivirus	86%	32%	5e-22
370	Contig55714	MNR	2063	Rhizoctonia solani endornavirus-RS002	85%	98%	0
371	Contig56398	MNR	1845	Rhizoctonia solani hypovirus 10	99%	89%	0
372	Contig57083	MNR	246	Riboviria sp.	90%	44%	4e-13
373	Contig57573	MNR	343	Ceratobasidium endornavirus 2	90%	50%	3e-28
374	Contig57952 [a]	MNR	263	Lentinula edodes tymo-like virus 1	84%	47%	8e-13
375	Contig59305	MNR	328	Rhizoctonia solani endornavirus-RS006-2	99%	93%	5e-65
376	Contig59316 [a]	MNR	566	Rhizoctonia solani negative-stranded virus 1	92%	43%	2e-36
377	Contig59449	MNR	1259	Rhizoctonia solani ourmia-like virus 5	90%	42%	4e-69
378	Contig594991 [a]	MNR	391	Rhizoctonia solani negative-stranded virus 3	99%	91%	2e-75
379	Contig59633	MNR	501	Rhizoctonia solani fusarivirus 4	98%	98%	3e-116
380	Contig60033 [a]	MNR	937	Rhizoctonia solani negative-stranded virus 3	81%	47%	9e-64
381	Contig60206	MNR	583	Sclerotinia sclerotiorum ourmia-like virus 9	79%	34%	2e-15
382	Contig61133	MNR	742	Ceratobasidium endornavirus C	89%	34%	1e-37
383	Contig61770	MNR	814	Binucleate Rhizoctonia hypovirus 1	99%	67%	2e-115
384	Contig62097	MNR	583	Binucleate Rhizoctonia hypovirus 1	99%	100%	3e-129
385	Contig63192	MNR	744	Black grass cryptic virus 2	99%	45%	6e-58
386	Contig63193	MNR	682	uncultured Partitiviridae sp.	88%	44%	6e-53
387	Contig63294	MNR	289	Rhizoctonia solani fusarivirus 3	99%	47%	2e-18
388	Contig63295	MNR	575	Rhizoctonia solani fusarivirus 3	99%	69%	3e-70

389	Contig63368	MNR	536	Rhizoctonia solani endornavirus-RS058-1	84%	43%	1e-34
390	Contig1002	MNR	376	Rhizoctonia solani mitovirus 106	98%	61%	6e-32
391	Contig63710	MNR	1429	Ceratobasidium endornavirus C	81%	39%	4e-73
392	Contig64177	MNR	662	uncultured partitivirus	28%	47%	2e-07
393	Contig64768	MNR	354	Sarcosphaera coronaria partitivirus	99%	75%	9e-53
394	Contig67201	MNR	972	Rosellinia necatrix partitivirus 7	92%	52%	6e-96
395	Contig67587	MNR	380	Armillaria mellea ourmia-like virus 1	86%	42%	5e-06
396	Contig67619	MNR	358	Nemesia ring necrosis virus	89%	36%	3e-10
397	Contig67802	MNR	1332	Erysiphales narna-like virus 3	82%	34%	6e-50
398	Contig68232	MNR	940	Rhizoctonia solani fusarivirus 3	99%	53%	4e-104
399	Contig68353	MNR	494	Rosellinia necatrix partitivirus 24	99%	64%	1e-67
400	Contig69027	MNR	860	Erysiphe necator associated ourmia-like virus 3	54%	36%	4e-16
401	Contig69619 [a]	MNR	410	Bat tymo-like virus	95%	41%	1e-20
402	Contig70096	MNR	1771	Rhizoctonia solani fusarivirus 3	91%	56%	1e-167
403	Contig70107	MNR	534	Sclerotium hydrophilum virus 1	82%	80%	2e-72
404	Contig70128	MNR	359	Burdock mottle virus	62%	53%	4e-12
405	Contig72127	MNR	1385	Morchella importuna endornavirus 1	95%	49%	6e-126
406	Contig72133	MNR	420	Burdock mottle virus	81%	46%	2e-21
407	Contig72364	MNR	362	Rhizoctonia solani fusarivirus 3	99%	60%	4e-41
408	Contig72925	MNR	427	Sclerotium rolfsii beny-like virus 1	84%	35%	3e-10
409	Contig72993	MNR	982	Rhizoctonia solani fusarivirus 3	98%	32%	4e-29
410	Contig73098	MNR	868	Rhizoctonia solani bunya/phlebo-like virus 1	90%	29%	7e-16
411	Contig73195	MNR	290	Rhizoctonia solani endornavirus-RS024-1	99%	99%	3e-61
412	Contig73448	MNR	886	Rhizoctonia solani ourmia-like virus 5	92%	49%	1e-65
413	Contig74745	MNR	413	Rosellinia necatrix partitivirus 19	90%	57%	1e-39
414	Contig74863	MNR	316	Rhizoctonia solani endornavirus 6	98%	88%	1e-57

415	Contig75133	MNR	250	Rhizoctonia solani fusarivirus 4	99%	98%	2e-46
416	Contig75191	MNR	825	Grapevine associated tymo-like virus	88%	32%	6e-24
417	Contig75269	MNR	466	Rhizoctonia solani partitivirus virus 4	72%	65%	4e-49
418	Contig75418	MNR	296	Endornavirus-like virus	97%	44%	5e-15
419	Contig2480	MNR	369	Rhizoctonia solani mitovirus 33	99%	75%	2e-58
420	Contig75710	MNR	578	Apple ourmia-like virus 3	99%	45%	9e-40
421	Contig75863	MNR	582	Ceratobasidium endornavirus B	94%	28%	7e-08
422	Contig76352	MNR	832	Sclerotium hydrophilum virus 2	92%	71%	2e-131
423	Contig77123	MNR	410	Rhizoctonia solani fusarivirus 3	98%	77%	1e-65
424	Contig77370	MNR	555	Beet necrotic yellow vein virus	73%	38%	2e-19
425	Contig77486	MNR	369	Rhizoctonia solani endornavirus 1	96%	54%	7e-35
426	Contig77550	MNR	271	Maize chlorotic mottle virus	98%	100%	1e-56
427	Contig77557	MNR	841	Diuris pendunculata cryptic virus	62%	31%	1e-17
428	Contig78826	MNR	651	Rhizoctonia solani fusarivirus 3	100%	81%	2e-106
429	Contig79475 [a]	MNR	221	Rhizoctonia solani negative-stranded virus 1	99%	88%	3e-36
430	Contig79687	MNR	299	Passion fruit yellow mosaic virus	97%	33%	8e-06
431	Contig80150	MNR	333	Rhizoctonia solani mitovirus 29	97%	56%	7e-27
432	First_Contig830	MNR	326	Macrophomina phaseolina mitovirus 3	98%	55%	7e-29
433	Contig80280	MNR	702	Rhizoctonia solani endornavirus 1	99%	33%	4e-24
434	Contig81423 [a]	MNR	263	Rhizoctonia solani negative-stranded virus 3	98%	58%	9e-25
435	Contig81873	MNR	259	Rhizoctonia solani partitivirus virus 4	97%	64%	6e-31
436	Contig82133 [a]	MNR	509	Agaricus bisporus virus 12	99%	29%	1e-06
437	Contig82475	MNR	209	Sanya totivirus 4	77%	46%	5e-09
438	Contig82498	MNR	1089	Diatom colony associated dsRNA virus 17 genome type A	87%	34%	3e-44
439	Contig83123	MNR	476	Rhizoctonia solani fusarivirus 4	99%	80%	3e-70
440	Contig83592	MNR	1038	Rhizoctonia solani fusarivirus 3	99%	68%	4e-143

441	Contig83859	MNR	952	Rhizoctonia solani fusarivirus 3	99%	67%	3e-130
442	Contig84441	MNR	529	Agaricus bisporus virus 11	90%	52%	3e-37
443	Contig84828	MNR	648	Grapevine-associated mymona-like virus 1	83%	33%	2e-18
444	Contig85382	MNR	366	Sclerotium hydrophilum virus 1	94%	81%	4e-60
445	Contig85594	MNR	731	Rhizoctonia solani fusarivirus 3	96%	41%	2e-44
446	Contig85804	MNR	656	Rhizoctonia solani fusarivirus 4	99%	99%	3e-147
447	Contig86139	MNR	305	Alphaendornavirus sp.	92%	55%	7e-30
448	Contig86329	MNR	304	Gaeumannomyces tritici partitivirus 1	59%	52%	8e-11
449	Contig86830	MNR	403	Rosellinia necatrix partitivirus 19	99%	76%	3e-63
450	Contig87038	MNR	620	Beihai razor shell virus 4	96%	31%	2e-14
451	Contig87694	MNR	421	Elderberry carlavirus B	89%	32%	3e-04
452	Contig87744	MNR	430	Sclerotium rolfsii hypovirus 8	99%	58%	2e-37
453	Contig88132	MNR	278	Heterobasidion partitivirus 4	82%	67%	7e-27
454	Contig88726	MNR	931	Rhizoctonia solani fusarivirus 3	99%	85%	5e-173
455	Contig88830 [a]	MNR	1235	Soybean leaf-associated negative-stranded RNA virus 4	98%	32%	2e-58
456	Contig88839	MNR	594	Rhizoctonia solani fusarivirus 4	99%	97%	8e-127
457	Contig89069 [a]	MNR	279	Magnaporthe oryzae mononegaambi virus 1	90%	40%	2e-11
458	Contig89150	MNR	354	Rhizoctonia solani endornavirus-RS002	51%	59%	1e-12
459	Contig90053	MNR	206	Rhizoctonia solani fusarivirus 4	99%	97%	4e-37
460	Contig90123	MNR	465	Rhizoctonia solani endornavirus-RS058-1	72%	53%	2e-34
461	Contig90626	MNR	671	Rhizoctonia solani negative-stranded virus 1	99%	57%	2e-74
462	Contig91173	MNR	665	Rhizoctonia solani ourmia-like virus 5	99%	49%	3e-54
463	Contig91694	MNR	249	Rhizoctonia solani fusarivirus 4	97%	100%	3e-47
464	Contig91788	MNR	514	Trichoderma harzianum hypovirus 1	95%	27%	3e-08
465	Contig91834	MNR	554	Rhizoctonia solani endornavirus-RS002	99%	99%	1e-75
466	Contig92293	MNR	293	Rhizoctonia solani fusarivirus 3	99%	93%	1e-54

467	Contig92338	MNR	681	Rhizoctonia solani hypovirus 1	98%	68%	8e-94
468	Contig6498	MNR	303	Rhizoctonia solani mitovirus 14	96%	47%	2e-20
469	Contig93348	MNR	300	Sclerotinia sclerotiorum mycoalphavirus virus 1	94%	37%	3e-20
470	Contig94125	MNR	337	Rhizoctonia solani fusarivirus 3	98%	79%	3e-56
471	Contig94158	MNR	253	Helicobasidium mompa dsRNA mycovirus	80%	68%	7e-22
472	Contig94210	MNR	627	Rhizoctonia solani fusarivirus 2	99%	62%	4e-87
473	Contig94389	MNR	491	Sclerotium rolfsii hypovirus 8	89%	51%	7e-42
474	Contig95180	MNR	377	Rhizoctonia solani endornavirus 2	99%	44%	2e-27
475	Contig4724	MNR	297	Rhizoctonia solani mitovirus 4	90%	69%	3e-34
476	Contig95612	MNR	394	Rhizoctonia solani endornavirus-RS058-1	99%	96%	3e-88
477	Contig118767	MNR	292	Rhizoctonia solani mitovirus 28	98%	42%	2e-08
478	Contig96241	MNR	557	Rhizoctonia solani endornavirus-RS098-1	99%	97%	6e-129
479	Contig96851 [a]	MNR	940	Rhizoctonia solani negative-stranded virus 1	90%	76%	1e-141
480	Contig97781	MNR	219	Fusarium solani partitivirus 2	97%	62%	2e-23
481	Contig97838	MNR	244	Rhizoctonia solani endornavirus-RS058-1	98%	94%	3e-45
482	Contig98258	MNR	412	Rhizoctonia solani endornavirus 5	99%	54%	5e-40
483	Contig98390	MNR	934	Rhizoctonia solani partitivirus 8	88%	60%	2e-110
484	Contig98883 [a]	MNR	261	Sclerotinia sclerotiorum mycotymovirus 1	98%	42%	3e-11
485	Contig98890	MNR	582	Plasmopara associated mycobunyavirales-like virus 2	91%	34%	5e-15
486	Contig100343	MNR	267	Agaricus bisporus virus 13	97%	43%	4e-10
487	Contig100624 [a]	MNR	349	Rhizoctonia solani negative-stranded virus 2	95%	83%	5e-27
488	Contig100862 [a]	MNR	523	Penicillium cairnsense negative-stranded RNA virus 1	96%	29%	2e-13
489	Contig101162	MNR	259	Rosellinia necatrix partitivirus 22	99%	79%	2e-43
490	Contig101165 [a]	MNR	298	Rhizoctonia solani negative-stranded virus 1	99%	60%	2e-31
491	Contig101452	MNR	652	Rhizoctonia solani fusarivirus 3	99%	87%	8e-116
492	Contig101748	MNR	626	Ceratobasidium endornavirus C	99%	57%	6e-76

493	Contig103956	MNR	282	Bipolaris maydis partitivirus 1	97%	57%	9e-25
494	Contig104147	MNR	393	Rosellinia necatrix partitivirus 7	99%	89%	3e-75
495	Contig104192	MNR	369	Lentinula edodes tymo-like virus 1	100%	44%	2e-24
496	Contig104202	MNR	448	Rhizoctonia solani fusarivirus 2	97%	36%	2e-20
497	Contig104383 [a]	MNR	412	Rhizoctonia solani negative-stranded virus 2	99%	63%	4e-46
498	Contig104432	MNR	836	Rhizoctonia solani fusarivirus 2	99%	61%	8e-109
499	Contig104487 [a]	MNR	334	Rhizoctonia solani negative-stranded virus 2	98%	65%	2e-42
500	Contig104495	MNR	319	Cryphonectria hypovirus 1	97%	42%	2e-14
501	Contig104697	MNR	509	Rhizoctonia fumigata mycovirus	99%	47%	2e-34
502	Contig104854	MNR	440	Rhizoctonia solani fusarivirus 1	86%	52%	7e-33
503	Contig104959	MNR	837	Rosellinia necatrix partitivirus 7	89%	76%	2e-129
504	Contig105365	MNR	554	Sclerotium rolfsii hypovirus 8	99%	44%	8e-31
505	Contig105698	MNR	300	Rhizoctonia solani endornavirus 6	99%	85%	5e-39
506	Contig105996 [a]	MNR	335	Rhizoctonia solani negative-stranded virus 3	99%	82%	4e-58
507	Contig106094	MNR	332	Rosellinia necatrix hypovirus 1	63%	38%	2e-10
508	Contig106149	MNR	573	Rhizoctonia solani partitivirus 7	95%	58%	5e-63
509	Contig106356	MNR	283	Rhizoctonia solani endornavirus-RS002	99%	96%	8e-53
510	Contig106552	MNR	424	Rhizoctonia solani fusarivirus 3	99%	48%	7e-37
511	Contig106837	MNR	446	Rhizoctonia solani alphavirus-like 4	95%	64%	8e-55
512	Contig106918	MNR	402	Rhizoctonia solani fusarivirus 3	98%	55%	7e-41
513	Contig107085	MNR	303	Rosellinia necatrix partitivirus 2	97%	40%	5e-12
514	Contig107789	MNR	387	Sclerotium rolfsii hypovirus 8	92%	51%	8e-19
515	Contig107812 [a]	MNR	351	Soybean leaf-associated negative-stranded RNA virus 4	98%	29%	2e-08
516	Contig107956 [a]	MNR	377	Rhizoctonia solani negative-stranded virus 2	81%	45%	3e-16
517	Contig108297	MNR	739	Rhizoctonia solani fusarivirus 3	56%	39%	3e-34
518	Contig108745	MNR	255	Rhizoctonia solani partitivirus 1	97%	45%	2e-1

519	Contig108777 [a]	MNR	412	Rhizoctonia solani negative-stranded virus 1	99%	62%	2e-39
520	Contig109012	MNR	363	Heterobasidion partitivirus 15	100%	54%	3e-31
521	Contig109045	MNR	380	Heterobasidion partitivirus 4	92%	57%	3e-38
522	Contig109562	MNR	355	Fusarium solani partitivirus 2	55%	47%	2e-13
523	Contig109737 [a]	MNR	302	Soybean leaf-associated negative-stranded RNA virus 4	99%	37%	3e-09
524	Contig109804	MNR	384	Plasmopara viticola associated Partitivirus 9	96%	59%	7e-37
525	Contig109942	MNR	268	Rhizoctonia fumigata mycovirus	96%	54%	1e-22
526	Contig110495	MNR	663	Agaricus bisporus virus 13	93%	49%	2e-58
527	Contig110648	MNR	493	Agaricus bisporus virus 13	93%	50%	6e-36
528	Contig110721	MNR	236	Endornavirus-like virus	82%	40%	1e-09
529	Contig111317	MNR	534	Rhizoctonia solani fusarivirus 3	96%	78%	8e-83
530	Contig111570 [a]	MNR	406	Rhizoctonia solani negative-stranded virus 2	98%	57%	2e-41
531	Contig112116 [a]	MNR	243	Rhizoctonia solani negative-stranded virus 3	98%	84%	8e-39
532	Contig112215 [a]	MNR	276	Rhizoctonia solani negative-stranded virus 2	95%	63%	4e-28
533	Contig112455	MNR	329	Rhizoctonia solani fusarivirus 2	94%	38%	9e-16
534	Contig113163	MNR	267	Lentinula edodes deltaflexivirus 2	98%	65%	3e-15
535	Contig113323	MNR	259	Rhizoctonia solani mycovirus 2	99%	65%	6e-32
536	Contig113765	MNR	254	Ceratobasidium endornavirus C	99%	63%	1e-28
537	Contig113919	MNR	454	Ceratobasidium endornavirus C	99%	37%	3e-19
538	Contig114427	MNR	341	Rhizoctonia solani mycovirus 2	99%	65%	2e-48
539	Contig114705	MNR	319	Rhizoctonia solani RNA virus 1	98%	82%	6e-38
540	Contig115831	MNR	345	Neofusicoccum luteum fusarivirus 1	95%	45%	2e-23
541	Contig116509	MNR	331	Sanya totivirus 2	95%	52%	4e-20
542	Contig116782	MNR	214	Ceratobasidium virus B	77%	60%	1e-13
543	Contig116811	MNR	244	Rhizoctonia solani endornavirus-PR-2	99%	49%	2e-16
544	Contig116950	MNR	305	Beihai sesarmid crab virus 7	91%	46%	5e-15

545	Contig117655	MNR	468	Ceratobasidium endornavirus C	98%	38%	5e-31
546	Contig117759	MNR	252	Rhizoctonia solani endornavirus-PR-2	73%	58%	3e-14
547	Contig95982	MNR	255	Rhizoctonia solani mitovirus 7	98%	77%	2e-35
548	Contig118773	MNR	541	Rhizoctonia solani endornavirus-RS002	99%	99%	9e-120
549	Contig119100	MNR	288	Rhizoctonia solani fusarivirus 3	97%	79%	3e-44
550	Contig119204	MNR	309	Rhizoctonia solani beny-like virus 1	98%	62%	1e-31
551	Contig119314	MNR	308	Grapevine Red Globe virus	97%	43%	1e-14
552	Contig119411	MNR	511	Rosellinia necatrix partitivirus 7	98%	52%	2e-52
553	Contig119458	MNR	480	Fusarium poae fusarivirus 1	95%	34%	3e-20
554	Contig119833	MNR	336	Rhizoctonia solani fusarivirus 2	98%	72%	2e-50
555	Contig119893	MNR	328	Rosellinia necatrix fusarivirus 1	69%	45%	6e-13
556	Contig120001	MNR	216	Vicia faba partitivirus 1	77%	59%	1e-10
557	Contig120070	MNR	266	Grapevine fleck virus	89%	45%	1e-09
558	Contig120076	MNR	606	Rhizoctonia solani virus-RS002	94%	44%	1e-43
559	Contig120227	MNR	479	Endornavirus-like virus	61%	47%	8e-08
560	Contig120356	MNR	216	Rhizoctonia solani endornavirus-RS002	97%	100%	5e-29
561	Contig120390	MNR	378	Callinectes sapidus toti-like virus 1	92%	45%	5e-26
562	Contig75492	MNR	247	Rhizoctonia solani mitovirus 16	98%	67%	5e-29
563	Contig120709	MNR	245	Rhizoctonia solani dsRNA virus 1	97%	57%	3e-22
564	Contig121363	MNR	283	Rhizoctonia solani endornavirus-RS006-2	98%	55%	3e-28
565	Contig121523	MNR	429	Ceratobasidium hypovirus A	80%	49%	2e-26
566	Contig121532	MNR	342	Rhizoctonia solani fusarivirus 3	100%	88%	5e-64
567	Contig122456	MNR	271	Piper methysticum tymovirus 1	79%	54%	2e-12
568	Contig122757	MNR	517	Rhizoctonia solani hypovirus 1	99%	34%	6e-22
569	Contig122870	MNR	376	Rhizoctonia solani endornavirus-RS002	94%	42%	2e-23
570	Contig122901	MNR	422	Rhizoctonia solani endornavirus 5	98%	54%	1e-42

571	Contig123125	MNR	268	Rose partitivirus	98%	64%	9e-29
572	Contig123349	MNR	574	Beihai razor shell virus 4	58%	33%	7e-07
573	Contig123716	MNR	360	Rhizoctonia solani endornavirus-RS006-2	95%	44%	2e-23
574	Contig123752	MNR	368	Ceratobasidium endornavirus C	99%	64%	1e-43
575	Contig124447 [a]	MNR	401	Rhizoctonia solani negative-stranded virus 1	98%	62%	3e-51
576	Contig124506	MNR	227	Rhizoctonia solani fusarivirus 3	98%	82%	6e-16
577	Contig124553	MNR	255	Rhizoctonia solani fusarivirus 2	98%	62%	1e-28
578	Contig125132	MNR	357	Ceratobasidium mycovirus-like	96%	50%	3e-26
579	Contig125714	MNR	226	Rhizoctonia solani fusarivirus 3	98%	89%	2e-39
580	Contig126004	MNR	255	Rhizoctonia solani endornavirus 1	100%	58%	6e-28
581	Contig63584	MNR	220	Rhizoctonia solani mitovirus 22	99%	68%	7e-25
582	Contig126523	MNR	506	Rhizoctonia solani alphaendornavirus 1	97%	45%	6e-38
583	Contig126748	MNR	220	Entomophthora benyvirus E	95%	45%	7e-12
584	Contig126894	MNR	303	Sinomenium acutum tymovirus 1	99%	47%	2e-24
585	Contig126965	MNR	236	Rhizoctonia solani alphaendornavirus 1	97%	51%	2e-17
586	Contig127528	MNR	342	Rosellinia necatrix fusarivirus 1	100%	60%	4e-34
587	Contig127771 [a]	MNR	266	Rhizoctonia solani negative-stranded virus 7	99%	100%	8e-52
588	Contig129154	MNR	277	Rhizoctonia solani endornavirus-RS024-1	98%	93%	1e-50
589	Contig129402	MNR	215	Rhizoctonia solani endornavirus 6	89%	84%	3e-13
590	Contig129690	MNR	209	Rhizoctonia solani endornavirus-RS058-1	84%	56%	5e-13
591	Contig126417	MNR	207	Binucleate Rhizoctonia mitovirus K1	98%	85%	6e-34
592	Contig130373	MNR	218	Medicago sativa alphapartitivirus 1	99%	68%	7e-25
593	Contig130919	MNR	261	Sinomenium acutum tymovirus 1	98%	47%	4e-14

Note: ‘[a]’ indicated the unclassified putative mycoviruses found in BNR or MNR in this study using metatranscriptome sequencing.
